# Analysis of Android Device-Based Solutions for Fall Detection

**DOI:** 10.3390/s150817827

**Published:** 2015-07-23

**Authors:** Eduardo Casilari, Rafael Luque, María-José Morón

**Affiliations:** Departamento de Tecnología Electrónica, ETSI Telecomunicación, Universidad de Málaga, 29071 Málaga, Spain; E-Mails: rluque@uma.es (R.L.); mjmoron@uma.es (M.-J.M.)

**Keywords:** fall detection, smartphone, eHealth, Android, accelerometer

## Abstract

Falls are a major cause of health and psychological problems as well as hospitalization costs among older adults. Thus, the investigation on automatic Fall Detection Systems (FDSs) has received special attention from the research community during the last decade. In this area, the widespread popularity, decreasing price, computing capabilities, built-in sensors and multiplicity of wireless interfaces of Android-based devices (especially smartphones) have fostered the adoption of this technology to deploy wearable and inexpensive architectures for fall detection. This paper presents a critical and thorough analysis of those existing fall detection systems that are based on Android devices. The review systematically classifies and compares the proposals of the literature taking into account different criteria such as the system architecture, the employed sensors, the detection algorithm or the response in case of a fall alarms. The study emphasizes the analysis of the evaluation methods that are employed to assess the effectiveness of the detection process. The review reveals the complete lack of a reference framework to validate and compare the proposals. In addition, the study also shows that most research works do not evaluate the actual applicability of the Android devices (with limited battery and computing resources) to fall detection solutions.

## 1. Introduction

The health, social, psychological and economic consequences of falls in older people has been a constant research topic of medical concern for the last three decades [1,2]. According to different epidemiologic analysis from the World Health Organization [3,4], a noteworthy fraction of the population (28%–35%) older than 64 undergoes at least a fall every year. In the USA, for example, an annual fall incidence of 50% [5] (with 9% enduring severe injuries) has been reported among the residents in nursing homes, while a fall rate ranging between 14.7% and 34% per annum has been measured for the older population of five different Asian countries [6]. Besides, falls constitute one of the leading causes of mortality among American older patients [5], provoking 40% of all deaths by injuries [3]. In 2013, about 25,500 American older adults passed away from fall related injuries [7]. In the case of the European Union falls provoked the death of 34,400 persons older than 60 in the period 2008–2010 [8]. Similarly, the Global Burden of Disease and Injury Study estimated 540,499 deaths caused by falls all around the world (with more than 115,000 victims aged over 65) in 2010 [9].

The relevance of the health problems connected to falls becomes more evident if we consider the worldwide growth of the life expectancy. An increasing proportion of elderly people (especially in the societies of Western countries) daily face the hazards of living on their own. 20%–30% of older people experiencing a fall suffer moderate to severe bruises, hip fractures or head traumas [10]. The injuries accompanying falls normally have severe aftereffects: after a hip fracture, 50% of older people are incapable of developing an independent living, 25% pass away within six months while 33% die within one year [11]. As a matter of fact, the global mortality rate from falls among older Americans augmented by 55% during the period 1999–2007 [12]. In 2010 falls accounted for more than 85% of years lived with disability (YLDs) provoked by unintentional injuries (excluding traffic accidents) in adults aged over 69 [13].

In this sense, falls are not only the main cause of hospitalization but also a major source of fear and loss of independence among the older adults. Up to 1% of falls among older people result in a hip fracture [14], but even moderate fall injuries can provoke an acute voluntary diminishment of the patients’ mobility as well as intense psychological disorders. In fact, the Fear Of Falling (FOF) has been recognized as a specific syndrome of the older people, typically related to an increase of anxiety and neuroticism [15]. FOF may in turn induce a fall recurrence, as previous fallers have been found to experience a probability of more than 60% of suffering a new fall during the year following the first accident [14].

Already in 1998, a study [9] reported that the injuries derived from a typical fall of an older adult entailed an average sanitary cost of more than $19,000 (comprising nursing home, emergency room and hospital- or home- health care but excluding doctors’ services). Stevens *et al.* in [16] have evaluated that the direct medical expenses caused by falls among American old people exceed $20 billion in 2010. These costs are forecast to rocket during the next years, as the annual direct and indirect costs of fall-induced injuries are estimated to reach $67.7 billion by 2020 [17].

It has been largely proved that the delay of the medical intervention after an accident is strongly linked to the morbidity and mortality rates from fall [18]. 50% of those who suffered a period of lying on the floor longer than 1 h died before six months after the collapse [19]. Thus, the development of trustworthy and economically sustainable systems for fall detection and emergency assistance notifications is crucial to guarantee not only an adequate medical response in case of falls but also to keep the standards of quality of life of the elderly.

A cost-effective and easy-to-deploy way of implementing fall detector systems is to reuse the sensing and computing capabilities of Android personal devices, which are currently omnipresent in the daily life of citizens. Obviously, the penetration of smartphones and Android technology among older people is lower than in other age groups. However, at least in Western countries, this situation is rapidly changing. In [20], Deloitte predicted that the technological gap between generations (as it refers to the smartphone penetration) will narrow over the following year to become negligible by 2020. Facts seem to confirm that projection, as in just one year (from May 2013 to May 2014), Deloitte has reported an increase of ten percentage points (from 40% to 50%) in the use of smartphones among British people aged over 55 [21]. The results from a questionnaire described in the same document reveal than people older than 65 consult their smartphones 13 times per day on average. Similarly, a recent tracking survey of Pew Research Center informed that only 23% of American seniors above 65 did not utilize cell phones in 2012 [22]. Although the adoption patterns of technological devices among older people strongly depend on the age, even among the oldest American seniors (those over 80) the use of cell phones sat at 61% in 2012 (with a percentage of 84% of users among those aged between 65 and 69). The use of smartphones among seniors in the USA (although still relatively low) is also growing at a remarkable rate, rising from 18% in 2012 [22] to 27% in 2014 [23]. Thus, we can project that mobile phones will become a daily and familiar tool for a notable proportion of the elderly population in developed countries in less than 10 years.

The smartphone usage and barriers among the elderly has been recently studied by different works. Mohadisdudis [24] has highlighted that the reluctance to smartphone is the result of a combination of factors (mainly due to economical limitations, vision impairments and lack of interest in technology). Concerning this matter, emergency calls and alarms are quoted as one of the most attractive potentials of cell phones for the elderly in most sociological studies about smartphone use [25,26,27]. In fact, older people tend to contemplate mobile phones more as safety devices (or “lifeline”) than as interfaces for social communication [28]. Thus, home care, mobility and safety are central concepts in the way elderly perceive mobile phones. Preliminary results of the evaluation of several assistive technologies with real users (commented in [29]) show that fall detectors are favorably appreciated by older people. According to the results of the opinion poll presented in [23], when asked about their attitude towards their smartphones, 82% of smartphone-owning seniors described their phone as a liberating experience [23]. In this sense, the same study confirms that (as expected) seniors have a tendency to use their phones for a more limited number of applications than people from other age groups. Paradoxically, this can increase the attractiveness and feasibility of fall detection applications installed in seniors’ smartphones as FDS apps will not have to contend with other programs for the memory and computing resources of the devices. In this regard, Melander has given evidences in [30] that safety and security are linked to the usability and ergonomics of the technology (which are prioritized with respect to privacy issues). Therefore, we can presume that older people might exhibit a favorable attitude to fall detection systems if they feel that the system improves their safety while guaranteeing their freedom of movements in a seamless and automated way.

The goal of this paper is to provide an in-depth analysis of the existing detection systems that utilize Android. The survey does not only thoroughly reviews and categorizes the existing proposals but it also focuses on those practical aspects (consumption, coexistence with other running applications, selection of the metrics for the appraisal of the detection algorithm) which are normally neglected by the literature.

This paper is organized as it follows: after the introduction in this Section 1, Section 2 proposes a general classification of fall detection systems, paying special attention to the advantages of using Android devices and, in particular, Android smartphones (SPs). Section 3 revises the previous states-of-the-art on fall detection and describes the methodology that has been followed in this paper for the bibliographic search. Section 4 to Section 7 analyze in detail the existing research works on Android-based fall detectors from different perspectives. Thus, Section 4 organizes the proposed architectures depending on the typology and structure of the systems, focusing on the role that the Android device plays in each case. Section 5 studies the typology and parameterization of the algorithms that are employed to carry out the fall detection. Section 6 surveys the different responses that the analyzed detection systems present when a fall is detected. Section 7 in turn portrays the procedures, experimental testbeds and metrics that have been utilized to evaluate the effectiveness of fall detections systems. Finally, Section 8 summarizes the main conclusions of the work.

## 2. A Classification of Fall Detection Systems (FDSs): Advantages of Smartphones

Fall Detection Systems (FDSs) can be categorized into three general typologies [31]: vision based, ambient based and wearable device based approaches. Vision based detection systems employ cameras, placed at overhead positions, to track and characterize the user’s movement and asses the occurrence of falls. For this purpose, different techniques for image analysis have been proposed, such us shape modeling using spatiotemporal features, detection of the shape changes of the posture, 3D head position analysis, *etc*. Similarly, ambient based assistive systems are conventionally founded on the joint analysis of audiovisual signals together with other specific information (such as floor vibrational data or microphone signals) captured by environmental sensors. In this case, falls are identified by comparing the measured floor vibration and/or sound signals with a predefined set of patterns corresponding to diverse activities (walking, running, fall of small objects, *etc.*). In a recent work by Cheffena [32], an unusual fall detection system based on smartphone audio features is suggested. The system could be applicable in home environments where the phone is located in the vicinity of the monitored user.

Both vision and ambient based strategies (which can be cataloged as context-aware systems), as those presented in [33,34,35,36,37,38,39,40,41,42,43,44,45,46,47,48,49], result in several drawbacks. Firstly, the area where the user (or patient) is tracked is constrained to a particular monitoring zone (e.g., a set of rooms at the user’s home). Secondly, the installation, adjustment and maintenance of the hardware required by these systems may present a high cost. In fact, the adaptability of these systems to changes in the supervised area is quite reduced as they are carefully adjusted and parameterized for a very specific scenario. Besides, the accuracy of the fall detection may be strongly determined by external and non-controllable conditions, such as the illumination, the occurrence of audio artifacts [50] or the presence of sudden visual obstacles, which induce the existence of “blind” spots where the patient cannot be adequately tracked [51]. In addition, the users may be reluctant to the constant visual surveillance that these systems perform as they can feel the risk of having their privacy compromised (in this sense, context-aware architectures using motion sensing input devices, such as that presented in [49], which is based on the use of the Microsoft Kinnect depth sensor, can be considered much less intrusive).

On the other hand, FDSs based on wearable devices utilize the information captured by sensors (normally accelerometers) that are transported by the user as garments (or integrated in the clothes). Under this approach, the mobility of the user is not restricted to a constrained zone. Moreover, if the wearable devices are provided with a wide area wireless connection (e.g., a 3G/4G data interface), the system can track the patient almost ubiquitously. In this area, most Android fall detection architectures can be classified into this group of detection methodology.

In the pioneering works [52] on wearable detection systems that utilized 2G telephony, the cell phone was solely exploited as a simple (and hardware-limited) interface to the Internet (e.g., via GPRS). Nowadays Android smartphones integrate a wide array of movement and position sensors, ranging from accelerometers to digital compasses, GPS units or gyroscopes. During the last decade, smartphones have been incorporated as common personal devices in the everyday life of the users. Thus, when compared to a dedicated monitor device, cell phones reduce intrusiveness [53] as they are already permanently carried by many users, who contemplate them as an indispensable tool.

In this sense, over the past few years, the design of electronic systems have clearly shown a tendency to software product lines, which benefits from rapid software development by reusing coarse-grained components. Due to their widespread availability and their quickly reducing price, the election of smartphones as fall detection devices minimizes operational, constructional, distribution and installation costs. As a consequence, many smartphone-based fall detections systems have been proposed by the research literature during the last five years, while there is a decreasing number of new prototypes that are implemented on special-purpose hardware. In the same way, there are just a few examples of systems for fall detection employing specific Wireless Sensor Networks (WSN) with other transmission technologies (apart from those employed by smartphones). In [54,55,56], for example, authors present a prototype deployed with 802.15.4/ZigBee sensing motes. ZigBee is also considered as the transmission standard in the slip and fall detector/predictor prototype developed in [57]. The prototype is installed on a sensor board with a Jennic 5148 ZigBee module. An 802.15.4-compilant CC2420 transceiver is integrated in the wireless sensor mote presented by Pivato in [58]. In contrast with most studies, the consumption and lifetime of the mote is carefully studied. In [59] the accelerometer is connected to a specific wrist-worn ZigBee device. In [60] the sensed data from a triple axis accelerometer and a gyroscope (located at waist and ankles) are received via ZigBee by an agent module and then retransmitted to a mobile device. In this system human movements are classified with a clustering algorithm.

The functionality of fall detection has been also suggested for architectures of Body Area Networks (BANs) although they are not always finally deployed or evaluated. There are examples [61] of FDSs founded on general biometric Body Area Networks where different wearable sensors (pulse-oximeter, SpO_2_ or ECG sensors, scales, *etc.*) are integrated. CARA architecture [62] proposes to send the data of a wearable accelerometer to an external not-portable gateway which applies a thresholding method to asses if a fall occurs. Similarly, ZigBee is the wireless technology between the BAN and a central server in the system portrayed in [59]. In that system, Android handset devices can be utilized to remotely monitor the biosignals from the server as well as the fall alarms. Arduino Fio hardware (which contains a gyroscope and an accelerometer) is used as a compact wearable device to detect falls in [63]. In that architecture alarms are sent via Bluetooth to a nearby smartphone.

The review in [64] offers an extensive revision of commercial off-the-shelf wearable devices for fall detection. The most common position for these devices, which are normally provided with a “panic-button”, is the waist. Most products are powered by a lithium battery and employ an embedded acceleration sensor to detect falls.

### 2.1. Use of Other Mobile Operating Systems

Google’s Android is by far the most relevant Operating System (OS) in the market of smartphones, with a 78.0% share and more than 260 million shipped smartphones worldwide during the first quarter of 2015 [65].

Android is a Linux-based operating system conceived for touch screen mobile devices. Unlike Apple’s closed approach, under Android open platform, the programmer can reconfigure many significant hardware and software components in the devices (e.g., the accelerometer) as well as carry out a complete redesign of the user interface (which is key point when dealing with elderly-oriented applications).

As a result, Android is massively employed as the programming environment for most smartphone-based (SP-based) fall detection solutions that can be found in the literature. However, there are some works in the bibliography where the system is deployed on other mobile OS.

Initial SP-based FDSs were developed in (nowadays obsolete) Symbian OS [66] on Nokia phones [67]. In [68] (a work of 2011) a Java multiplatform software architecture (using an external accelerometer) is deployed in both a Symbian phone (Nokia 5800) and Android smartphones (Samsung Galaxy, HTC Hero) to detect falls. In the paper the detection system is not tested and the performance of these two operating systems is not compared.

Apple IOS is selected as the operating systems for the applications presented in [69], or [70], where an iPhone is responsible for warning the user about the fall risk based on the information of the signals received from different mobility sensors. The work in [70] develops an IOS Application running on an iPhone, which communicates with two Bluetooth low energy (BLE) modular sensors (containing 3-axis accelerometers, magnetometers and gyroscopes). The application is intended to automate the fall risk estimation for post stroke patients.

Majumder *et al.* present in [71] an iPhone-based application that receives and analyses the data sent (via Wi-Fi) by the accelerometers, pressure sensors and gyroscopes integrated in “smartshoes”. From these measurements, the application classifies the performed movements and warns the users about potential falls if any gait abnormality is detected. iOS is also utilized in the detector presented in [69].

The capability of the different mobile operating systems (in particular, Meego Harmattan, Symbian, Windows Phone, and Android) to develop applications for fall detection is discussed in [72]. From the performed analysis, authors conclude that Android constitutes the best election as long as it guarantees more programming support while it minimizes the programming and implementation time.

### 2.2. Other Uses of Android Smartphones in Personal Monitoring Systems for the Elderly

In the field of senior monitoring systems, Android smartphones have not only been employed as useful devices to detect falls. For example, SPs have been also proposed to track and assist dementia patients in their day-to-day life activity [73]. Fontecha presents in [74] a tracking system that makes use of a SP to obtain movement data related to gait and balance. From the analysis of these accelerometer data, the frailty risks of the elderly can be diagnosed. In other cases smartphones are used as a “holter” monitor or as a gateway of a Body Area Network consisting in a specific sensing platform with short range communications (e.g., Bluetooth). For example, the work in [75] presents an Arduino-based BAN that monitors the breathing activity and the heart rate. Data are sent via Bluetooth to a smartphone app, which decides if an alert text message must be sent

Tacconi develops in [76] an Android application that implements the Timed-Up-and-Go (TUG) test. This clinical test, which is commonly utilized to assess mobility: estimates the time required by an individual to stand up from a chair, walk 3 meters, return to the chair and sit down. [76]

SPs (combined with acceleration sensors and machine learning techniques) have also been proposed as wearable devices to detect Freezing of Gait (FoG) episodes in Parkinson patients [77]. Similarly, Android systems have been utilized in systems that do not detect falls but evaluate the fall risk. For example, in [78], Otis *et al.* present a prototype aimed at identifying the type of soil (a piece of information that can be useful for fall prevention schemes). The system consists of a shoe that incorporates a set of sensors and an Android SP that wirelessly receives and analyses in real-time the signals measured by the sensors. Authors suggest that the waveforms of the acceleration measured at the shoe can be employed to classify each type of ground. Android Smartphones have also been considered [79] in systems devoted to safety zone monitoring and wandering detection for people suffering dementia. Likewise, Guimaraes *et al.* utilize in [80] a SP-based Android platform to predict fall risks. By using the internal accelerometer in the SP, the developed program is in charge of measuring the step length, duration and velocity from acceleration signals during normal gait. In order to assess the importance of other fall risk factors, the SP in that system is also employed to administer questionnaires to the patients. An Android SP application for step monitoring system is presented and compared with a commercial pedometer in [81].

Android devices are used as simple panic button in [82] while the work in [83] describes a system to detect heart problems where an external heart beat sensor communicates with the SP via Bluetooth.

An important feature in SP-based applications aimed at human tracking is the capability of determining the position of the individual under supervision. Almost all current SPs are provided with GPS units. Consequently, the location provided by fall detectors and tracking system is conventionally based on GPS data. In very particular systems (planned for supervised indoor scenarios), the user location is achieved by triangulation of the RSSI of the signals received from different Wi-Fi Access Points [67].

## 3. Analysis of Android-Based Fall Detection Systems: States-of-the-Art and Bibliographic Search Methodology

The issues related to automatic fall detection have attracted many research efforts during the last decade. Thus, a vast literature on FDSs has been generated. The general principles of fall detection for elderly have been summarized by Yu [84] and Noury [18] while generic states-of-the-arts and different classifications of fall assessment techniques have been presented by Pannurat *et al.* [64], Hedge *et al.* [50], Noury *et al.* [18,85], Perry *et al.* [86], Patsadu [51], Hijaz *et al.* [87], El-Bendary *et al.* [88], Mubashir *et al.* [31], Igual *et al.* [29], Delahoz [89] and Abbate [90]. A systematic review of fall detectors based on body-worn sensors is also offered by Schwicker [91]. However, SP-based fall detection and prevention architectures are only specifically revised in great detail by Habib *et al.* in [92].

In this paper, we carry out an extensive and comparative study of the existing solutions to detect falls with any type of Android device. The review of the literature was performed by means of a thorough and systematic exploration of publically accessible electronic databases of peer-reviewed research sources (IEEE explorer, Scopus, Cochrane library and Ovid SP). The exploration strategy, which was restricted to English language publications, was mainly based on advanced text string searches in the title, abstract and keywords of the papers. The initial search terms were “Android” or “smartphone” combined with “fall” and “detection”, “prevention” or “prediction”. The direct exploration of the aforementioned databases was complemented by other search tools of scientific works (such as Pubget, Mendeley or Google Scholar) as well as by other informal document search engines (e.g., pdf finder). This Boolean analysis was repeated bimonthly until May 2015. In parallel, hand and cross reference search was performed.

After finding more than 500 papers that met the query criteria, we selected just those which describe a FDS where any kind of Android device (not only smartphones) was employed. The role of the Android device was not considered as a filtering criterion. Thus, although in most cases the Android devices were utilized as body-worn sensors, we also considered those architectures where Android elements are exploited as communication gateways or even just as final monitoring interfaces to receive alarms. On the other hand, those papers (mentioned in the previous section) dealing with systems merely aimed at assessing fall risk were excluded from the state-of-the-art (an exhaustive and comprehensive review of the literature on that topic can be found in [93]).

At the end of this bibliographical analysis, we found that 73 works included experimental results or pioneering research on Android-based fall detectors.

## 4. Analysis of the Typology, Role and Complexity of Android-Based Fall Detection Systems

In a first approximation to the nature of the proposals found in the literature, Table 1 describes the basic characteristics of the analyzed systems. The first column indicates the general typology of the architectures, which can be classified as Body-Worn (BWS) or Context-Aware Systems (CAS). As the table clearly shows, most architectures can be characterized as BWS, that is to say, body area networks that incorporate an Android device (typically a smartphone). This notwithstanding, there are also proposals where the Android device is part of a not wearable CAS. In addition there also exist systems where context aware and body-worn techniques are combined. For example, in order to analyze the user mobility and identify falls, the system described in [94] jointly makes use of the images captured by video cameras and data sensed by the accelerometer of a wearable Android platform.

The detection systems can also be cataloged depending on the role of the Android device. In the analyzed proposals, Android devices typically assume one (or a combination) of the following functionalities, which are indicated in Table 1.

- Sensor (S): the system exploits the sensing capabilities of the Android device. A specific column in Table 1 informs about the particular built-in sensor that is employed. In most architectures, the system exploits the tri-axis accelerometer that is embedded in the majority of existing SP models. To a lesser extent, the signals from embedded gyroscopes are also considered by some proposals. On the other hand, the same column in Table 1 also explicitly informs about those systems where external (not Android) sensors are utilized. The simultaneous utilization of the accelerometry signals captured by both an external (normally Bluetooth-enabled) sensor and a SP is proposed in works such as [95].
- Data Analyzer (DA): the system can benefit from the computing power in the Android platform to implement and execute the algorithm that determines if a fall has taken place. If the detection decision is based on the signals captured by external sensors, wired or (most preferably) wireless communication between the sensor and the Android device must be deployed.
- Communication Gateway (CG): according to this role, the communication interfaces (Wi-Fi, Bluetooth, GPRS/3G/4G, *etc.*) of the Android devices are employed to retransmit the sensed data (or the fall detection decision) to a remote central server.
- Remote Monitoring Unit (RMU): in that case the Android device (normally a SP or a tablet) is just integrated in the detection architecture as a final user interface to warn monitoring users (e.g., medical staff) about the fall occurrence. As Android SPs are typically provided with web browsers, SPs could be used in any FDS where falls are announced through a Web interface.

Table 1 does not include those works [55,56,59,96,97] where Android SPs are considered as specific RMUs (*i.e.*, with particularized apps to receive fall alarms). For example, the system in [96] connects a set of medical sensors via ZigBee to an Atmel board which in turns retransmit the biosignals (via Wi-Fi or UMTS) to an Android smartphone. Android phones are also operated as emergency interfaces in [97], where Kozlovszky *et al.* present a telemonitoring architecture to track the activity and biosignals of the elderly.

Apart from smartwatches, there are Android devices that are designed to capture different ergonomics data of the human mobility. Thus, MiMiC (Mobile Motion Capture) [98] is a wearable Android-programmable device that receives data from different kinematic sensors via Bluetooth. Authors propose to use MiMiC to detect falls although a detection system is really not implemented nor evaluated on the MiMiC board.

In some particular examples, SPs are merely employed to capture samples of the body acceleration in order to characterize the fall patterns. These samples are posteriorly and externally analyzed in an offline way by a computer. In other cases, just the roles as DA or CG are contemplated. However, as it can be concluded from the table, most solutions combine the three first possible roles of the Android devices, which simultaneously measure and appraise the mobility parameters of the monitored patient while performing as data gateways to Internet-connected monitoring points.

Due to the importance of SP-based systems, it is also worthwhile to characterize the way in which SPs are used. Thus, the fifth column in Table 1 discriminates three types of architectures:

- Smartphone-only or SP-only systems: those that integrate all the functionalities of the detection system (S, DA and/or CG) into a standalone app and a single Smartphone.
- “Combined” systems: those SP-based systems that require additional elements (such as external mobility sensors) to track the user.
- Specific Devices (SD): those architectures that do not contemplate the use of a smartphone and make use of an Android gadget or specialized Android hardware platform that has been purposely designed for movement tracking and/or fall detection. There are just a few examples of systems in the literature that can be included into this category, where we can also include the system described in [41], in which an Android smartwatch is employed as the mobility sensor.

### Discussion on the Quality of the Sensors Embedded in Android Devices

As aforementioned, the sensors embedded in commercial Android SPs are being massively employed by the wearable FDSs that have been proposed by the research literature over the last five years. In this area, an important issue still under discussion is the capability of these sensors (especially the built-in accelerometer) to fully characterize the mobility of the user that is transporting the SP. In many cases, the performance (an even the applicability) of a fall detection algorithm strongly relies on the quality of the installed sensors. SP-based detection systems have to deal with the variability in the resolution and the polling frequency of the accelerometry sensors that are embedded in the phones [99] but the fact is that in most papers the characteristics of the accelerometers are frequently not described. Moreover, some vendors of smartphones do not give details on the model or the features of the particular sensors that they integrate in their products. These sensors can even be changed over time by the manufacturer for the same smartphone model.

A limited analysis (based on a single example) of the capability of SP accelerometers to detect free falls has been presented by Vogt *et al.* in [100]. Authors conclude that a SP allows estimating the free-fall time with a good degree of accuracy. A more complete set of scenarios for the evaluation of fall sensors is proposed in [12].

The limited resolution and small range of built-in accelerometers have been considered as unsuitable to identify falls [101]. External accelerometers that are specifically used in combined approaches have a typical range between 6 g and 16 g. Conversely those integrated in smartphones hardly reach 2 g [101]. As a matter of fact, built-in accelerometers are just intended to recognize the orientation of the SP (horizontal or vertical) to settle the screen orientation. Thus, they are not conceived to offer an accurate characterization of the acceleration that the device experiences under any possible type of movement. Conversely, Mellone *et al*. in [102] systematically compared (with the movements of 59 subjects) the performance of the built-in accelerometer of a commercial SP with that of an accelerometry sensor specifically designed for motion and posture detection in clinical settings. As the results show no significant differences between the devices, the authors state that a SP can become a suitable tool for quantitative movement analysis with a clinical value. Albert *et al.* corroborated in [103] that the use of dedicated accelerometers in FDSs presents similar results to those obtained with smartphones. Moreover, the A/D (analog to digital) converters of built-in accelerometers in commercial smartphones employ 8 to 16 bits (typically 13 bits [104]) to represent the acceleration signal. Taking into account that the range is also normally limited to ±2 g, the typical resolution is below 0.001 g, which should not pose any special problem to the fall detection process.

Another crucial factor is the update frequency (or sampling rate) of the sensors. The impact of the sampling frequency of the accelerometer on the quality of the detection process is investigated in [105,106]. After comparing the performance of two threshold-based algorithms and different fall records, Fudickar *et al.* [106] conclude that fall detection with (low) sampling rates of at least 50 Hz can be enough to achieve a good performance (a sensitivity of 99%). Abbate also assumes in [107] that sampling at 50 Hz is a good trade-off between power consumption and accuracy to detect fall-like events.

The literature on Android-based FDSs does not usually give details on the way the sensors are configured. Android API (Application Programming Interface) enables the programmer to select the accelerometer sampling rate from four possible levels which are in turn defined by four predefined constants [108]: SENSOR_DELAY_NORMAL, SENSOR_DELAY_UI, SENSOR_DELAY_GAME and SENSOR_ DELAY_FASTEST. The actual rate set by these four levels (ranging from 7 Hz to 200 Hz) does not correspond to a fixed value as it strongly depends on the particular Android device that is being used. Furthermore, in the SP model employed in [105] (Samsung Galaxy Mini phones running Android version 2.2) Medrano *et al.* detected that the sampling rate was not stable. In that article authors propose a classifying system that requires a training phase with fall patterns. Taking into account that the system can be utilized in smartphones with a different sampling rate of that used to generate the training patterns, authors propose techniques such as subsampling or interpolation to mitigate this problem. In any case, these authors remark that current smart phone OSs such as Android and IOS impose heavy limitations regarding the configuration of specific sampling rates and the access of interruptions.

Besides, Lockhart *et al.* point out in [109] that current mobile devices should be modified to ease the development of applications for tracking and movement recognition. Smartphones were not conceived for continuous sensing and processing of data. For example, in many low-end Android smartphones, the sensors are not fully operative while the processor remains in a sleep mode, even if a background sensing application is being executed. Thus, the developers of a fall detecting system are obliged to implement a “wake lock” to prevent the processor from entering in a low consumption mode. However wake locks provoke an important increase in the battery drain, drastically reducing the lifetime of the battery.

**Table 1:** Analysis of the proposals: System typology, role and sensing characteristics of the SP.

Ref.	Year	General Typology: -Context Aware Systems (CAS) -Body-Worn System (BWS) -Combined (CAS and BWS)	Role of the Android Device: -Sensor (S) -Data analysis (DA) -Communication Gateway (CG)-	Number of Elements: -Smartphone-Only (SP-only) -SD (specific device) -Combined (SP and SD)	Employed Sensor(s)
[53]	2009	BWS	S, DA, CG	SP-only	Built-in tri-axis accelerometer
[82]	2010	BWS	S, DA, CG	SP-only	Built-in tri-axis accelerometer
[76]	2011	BWS	S, DA, CG	SP-only	Built in tri-axis accelerometer and orientation sensor
[99]	2011	BWS	S, DA, CG	SP-only	Built-in tri-axis accelerometer
[110] [111]	2010	BWS	S, DA, CG	Combined (SP and an external magnet)	Built-in tri-axis accelerometer (in [111] a magnetic sensor also used)
[112]	2010	BWS	S, DA, CG	SP-only	built-in tri-axis accelerometer and magnetometer
[113]	2011	BWS	CG	Combined	Specific Android based Personal Activity Monitor with accelerometer
[114]	2011	BWS	S, DA, CG	SP-only	Built-in tri-axis Bosch Sensortec’s 3-axis BMA150 accelerometer
[115]	2011	BWS	S, DA, CG	SP-only	Built-in tri-axis accelerometer
[116]	2011	BWS	S, DA, CG	SP-only	Built-in tri-axis accelerometer
[117] [118]	2011 2012	BWS	S, DA	SP-only	Built-in tri-axis accelerometer
[119]	2012	BWS	S, DA, CG	SP-only	Built-in tri-axis accelerometer
[120]	2012	BWS	S, DA	SP-only	Built-in tri-axis accelerometer
[121]	2012	BWS	S, DA	SP-only	Built-in tri-axis accelerometer
[122]	2012	BWS	S, DA, CG	Combined (external and internal sensors)	Built-in BMA150 3D accelerometer External 3-axis MMA7260Q accelerometer (in a Shimmer2 wireless sensor)
[123]	2012	CAS	S, DA, CG	SD	Doppler sensor in a Beagle Board-XM embedded computer
[124]	2012	BWS	S, DA, CG	SP-only	Built-in tri-axis accelerometer
[125]	2012	BWS	S, DA, CG	SP-only	Built-in tri-axis accelerometer
[103]	2012	BWS	S, DA	SP-only	Built-in tri-axis accelerometer
[126] [127]	2012	BWS	S, DA, CG	SP-only	Built-in tri-axis accelerometer
[128]	2012	BWS	S, DA, CG	SP-only	Built-in tri-axis accelerometer
[129]	2012	BWS	S	SP-only	Built-in tri-axis accelerometer and magnetometer
[130]	2012	BWS	CG	Combined (SP with an Arduino Board)	Arduino Duemilanove board with a ADXL335 tri-axis accelerometer and other medical sensors
[131]	2012	BWS	S, DA, CG	SP-only	Built-in accelerometer and orientation sensor
[132]	2012	BWS	S, DA, CG	SP-only	Built in accelerometer and orientation sensor
[133]	2012	BWS	S, DA, CG	SP-only	Built-in tri-axis accelerometer, gyroscope, and magnetic sensor
[134]	2012	BWS	DA, CG	Combined (SP and external accelerometer))	External tri-axis accelerometer ADXL345 of Analog Devices Inc. connected to a BT-enabled wearable unit
[61]	2013	BWS	CG	Combined	External Specific BT-enabled Body Activity Device) with a MXA2500 Dual Axis accelerometer
[135]	2013	BWS	S, DA, CG	SP-only	Built-in tri-axis accelerometer
[136]	2013	BWS	CG	Combined (external sensors)	Built-in tri-axis accelerometer of an external EZ430-Chronos
[55]	2013	BWS	S, DA	SP-only	Built-in BMA150 3D accelerometer, AK8973 and AK8973 orientation sensor,
[137]	2013	BWS	CG	Combined (external sensor)	TI SensorTag with an inertial unit, a barometer, and a temperature and humidity sensor
[138]	2013	BWS	RMU (Remote monitoring Unit)	SD	BT-enabled Embedded system provided with an accelerometer
[139]	2013	BWS	S, DA, CG	Combined (SP accelerometer and BT medical sensors)	Built-in tri-axis accelerometer (together with other Bluetooth enabled medical sensors)
[140]	2013	BWS	S, DA, CG	SP-only	Built-in tri-axis accelerometer
[141]	2013	BWS	S, DA	SD (WIMM, Android -based watch)	Built-in tri-axis accelerometer of a Smartwatch
[142]	2013	BWS	S, DA, CG	SP-only	Built-in tri-axis accelerometer and triaxial gyroscope
[143]	2013	Combined (BWS and bed presence detector)	DA, CG	Combined	BT and ZigBee enabled Specific ZigBee detector (belt) with STM LIS344ALH
[144]	2013	Combined (BWS and voice and image analysis)	S, DA, CG	SP-only device combined with external CAS system	Tri Built-in tri-axis accelerometer and external sensors: cameras and microphones
[145]	2013	BWS	CG	Combined (SP with an Arduino Board)	Arduino Duemilanove board with a ADXL335 tri-axis accelerometer and other medical sensors
[101]	2013	BWS	S, DA, CG,	SP-only	Built-in tri-axis accelerometer
[146]	2013	BWS	S, DA	SP-only	Built-in accelerometer and gyroscope
[147]	2013	BWS	S, DA, CG	SP-only	Built-in tri-axis accelerometer
[148]	2013	BWS	S, DA, CG	SP-only	Built-in tri-axis accelerometer, gyroscope, and magnetic sensor
[149]	2013	BWS	S, DA, CG	Combined (SP accelerometer and BT medical sensors)	Built-in tri-axis accelerometer (other BT-enabled medical sensors are integrated in the prototype to measures other biosignals)
[150]	2013	BWS	S, DA, CG	SP-only	Built-in tri-axis accelerometer
[151]	2013	BWS	S, DA, CG	SP-only	Built in accelerometer
[152]	2013	BWS	S, DA, CG	SP-only	Built-in tri-axis accelerometer
[72]	2013	BWS	S, DA, CG	SP-only	Built-in tri-axis accelerometer
[153] [154]	2013	BWS	S, DA	SP-only	Built-in tri-axis accelerometer and gyroscope
[94]	2014	Combined	Android sensor Platform (S)Android SP as a CG	Combined	Visual sensors and LilyPad tri-axis accelerometer
[69]	2014	BWS	S, DA	SP-only	Built-in accelerometer and gyroscope
[155]	2014	BWS	S, DA, CG	SP-only	Built-in tri-axis accelerometer
[105]	2014	BWS	S, CG	SP-only	Built-in tri-axis accelerometer
[156]	2014	BWS	S, DA, CG	SP-only	Built-in tri-axis accelerometer and gyroscope (electronic compass)
[157]	2014	BWS	S, DA, CG	SP-only	Built-in tri-axis accelerometer
[73]	2014	BWS	S, DA, CG	SP-only	Built-in tri-axis accelerometer
[95]	2014	BWS	DA, CG	Combined (SP and an external accelerometer)	BT-enabled TI eZ430-RF2560 device
[158]	2014	BWS	S, DA, CG	SP-only	Built-in tri-axis accelerometer
[159]	2014	BWS	S, DA, CG	SP-only	Built-in tri-axis accelerometer
[160]	2014	BWS	DA, CG	Combined (SP and BT-enabled smart watch)	built-in tri-axis accelerometer of a Smartwatch (Pebble Smart Watch)
[161]	2014	BWS	S, DA, CG	SP-only	Built-in tri-axis accelerometer
[162]	2014	BWS	S, DA	SP-only	Built-in tri-axis accelerometer, gyroscope and magnetometer
[163]	2014	BWS	S, DA	SP-only	Built-in tri-axis accelerometer and magnetometer
[164]	2014	BWS	S, DA, CG	SP-only	Built-in tri-axis accelerometer
[165]	2014	BWS	S, DA, CG	Combined (SP with an Arduino Board)	Built-in tri-axis accelerometer and external Freescale Board with a tri-axis accelerometer
[166]	2014	BWS	S, DA	SP-only	Built-in tri-axis accelerometer
[167]	2015	BWS	S, DA, CG	SP-only	Built-in tri-axis accelerometer
[168]	2015	BWS	S, DA, CG	SP-only	Built-in tri-axis accelerometer and gyroscope
[168]	2015	BWS	S, DA, CG	SP-only	Built-in tri-axis accelerometer and gyroscope
[169]	2015	BWS	S, DA	SP-only	Built-in tri-axis accelerometer and gyroscope
[170]	2015	BWS	S, DA, CG	SP-only	Built-in tri-axis accelerometer
[171]	2015	BWS	S, DA, CG	SP-only	Built-in tri-axis accelerometer
[172]	2015	BWS	S, DA, CG	SP-only	Built-in tri-axis accelerometer

## 5. Analysis of the Fall Detection Algorithms

A basic component in any FDS is the decision algorithm that is employed to distinguish fall events from other conventional movements, which are normally encompassed under the term “Activities of Daily Life” or ADLs. Computing techniques for fall classification can be coarsely classified into two broad categories [92]: Pattern Recognition Methods (PRM) and Threshold-Based Approaches (TBA).

Pattern recognition methods comprise Artificial Intelligence, rule-based and machine learning based algorithms that typically base on diverse classification techniques such as Neural Networks and perceptrons [36,167], instance based learning [173], fuzzy logic systems [33], Gaussian Mixture Model [174], decision trees [67], Naïve Bayes classifier [175], Hidden Markov Models [62,66], k-Nearest Neighbor [41], Fisher’s Discriminant Ratio [148], Hjorth parameters [176], k-mean [177] or Support Vector Machine [166].

Pattern recognition architectures normally imply high computational costs, massive analysis of data, accesses to databases and/or long training periods where the classification algorithm must be parameterized or adapted to the movement traces of a set of experimental users. In contrast, Threshold-Based approaches algorithms rely on the comparison between one or several magnitudes captured by the movement sensors (conventionally the module or one component of the acceleration vector) and certain decision thresholds.

Table 2 informs about the characteristics of the fall detection algorithms that are employed by the proposals in the literature. As it can be observed from the table, Android fall detection architectures are predominantly grounded on TBAs. Owing to the restrictions on computing and storage capabilities of most Android system, TBA are preferred as they can be straightforwardly implemented by a simple Android app, which can perform the threshold comparison in real-time.

Table 2 also itemizes the physical variables that are measured and considered by the systems to make the detection decision. In this sense, some studies [150] are focused on determining the most relevant variables that must be tracked and computed to detect a fall. El-Bendary *et al.* state in [88] that algorithms for fall detection that rely on a single “data provider” (accelerometer, camera, gyroscope, *etc.*) may impose severe limitations to ensure a high reliability. The study in [148] proposed a hierarchical classifying system to discriminate different standard mobility patterns. The paper investigated which features derived from the three embedded kinematic sensors of a SP (magnetometer, accelerometer and gyroscope) were more significant to the different considered mobility patterns. The results showed that the accelerometer coordinates are the most significant information to recognize physical activities, while the gyroscope and orientation sensors are more convenient to detect body posture and falls, respectively.

The initial position before the fall has been also investigated [132] as a key aspect in the evolution of the kinetics (acceleration, orientation) of the fall patterns. Due to the high number of variables that can be taken into account, a family of studies [132] is dedicated to investigate the proper election of the classifying features. In those cases, pattern recognition methods are finally proposed. The problem of these algorithms is the costly training phase that is necessary for a proper characterization of the impacting variables. As a consequence, as it can be verified in Table 2, many wearable systems take the module of the acceleration vector (or SMV, Signal Magnitude Vector) as the main (or unique) decision variable to be analyzed for the detection decision. This variable is defined as:

(1) $$SMV=\sqrt{A_{x}^{2}+A_{y}^{2}+A_{z}^{2}}$$

where *A_x_*, *A_y_* and *A_z_* are the acceleration components, that is to say, the readings in directions of *x*, *y*, and *z*-axis of the accelerometer that is transported by the monitored user.

Different metrics and statistics derived from these three components are also considered in other schemes.

### 5.1. Election of the Threshold

Obviously the election of an adequate threshold is a determining factor for the performance of a TBA algorithm. For each TBA-based proposal, Table 2 also specifies if the threshold is set to a fixed value or otherwise dynamically adjusted to the particular conditions of the monitored patient. In some studies, authors do not justify their (presumably heuristic) selection of the thresholds, while in other cases, they comment that the threshold value was settled after conducting a round of preliminary experiments. As a matter of fact, detection thresholds should be particularized for each patient [144]. The experiments developed by Cao [125] have shown that the performance of the TBA detection algorithms improves if the thresholds are set taking into account the characteristics of the user (e.g., the Body Mass Index or BMI). Medrano *et al.* in [161] investigate the effect of the personalization of the detection threshold among different kind of people. Although a personalized threshold reduces the number of false positives, authors conclude that the number of false alarms can be too high (more than one false positive per day is detected in a population of four subjects after three days of continuous monitoring of the ADLs). Nevertheless, the use of a simple kinetic threshold may leads to false alarms recurrently. Moreover, the use of a single threshold may not be suitable to detect different types of falls.

Besides, the position of the sensor strongly impacts on the performance. For example, the reference acceleration component heavily varies depending on the phone orientation. Kangas recommend in [178,179] combining simple TBA schemes with posture detection after the fall.

For those systems that are founded on the identification of patterns, Table 2 indicates if a training phase is required to tune the algorithm to the particular behavior of the individual under supervision. The work by [180] implemented and evaluated different algorithms to classify physical activities from data acquired with five small biaxial wearable accelerometers distributed on different parts of the body. The analysis showed that some activities can be adequately identified with independence of the subject but others seem to demand subject-specific training data.

An important drawback of requiring a training phase is that with both ADLs and fall patterns. It is not always evident to obtain a realistic sample of a body fall from the actual target user of the system (e.g., an old person). In those cases (as in the evaluation phase), simulated falls (normally performed by young and healthy individuals) are executed. The paper in [161] proposes a supervised technique to detect anomalies in the body balance. Accordingly, any deviation from normal movements is classified as a fall. Making so, the system can be trained without fall events.

### 5.2. Definition of a Fall and Discrimination of Fall Phases

The distinction between a body fall and an ordinary activity is normally unproblematic for the common human perception. A fall has been defined by the Kellogg international working group on the prevention of falls in the elderly as “unintentionally coming to ground, or some lower level not as a consequence of sustaining a violent blow, loss of consciousness, sudden onset of paralysis as in stroke or an epileptic seizure” ([181], quoted by [18]). In general, a fall can be described as a sudden and unintentional movement from an upright standing (or sitting) position to a reclining or almost horizontal posture. However, an accurate analytical definition of the concept of “fall” is not an easy task and is still an unresolved issue. An occasional abrupt alteration of a single physical magnitude associated to a patient’s movements cannot be straightforwardly associated to a fall occurrence. In point of fact, a fall is the result of a complex sequence of movements. In spite of the variable and irregular nature and typology of falls, this sequence has been decomposed into a series of typical “stages” or phases, which must be identified for a higher accuracy of the detection process. Thus, a fall, including the impact against the floor, is assumed to consist of four [85,106,140] or even five successive phases [101,157,182]:

1. The pre-fall, “idle” or “normal” [128] period, characterized by conventional Activities of Daily Living (ADLs) containing some signs of instability. Occasional actions originating unexpected movements (such as sitting or lying down rapidly) should be discriminated from a fall.
2. The free-fall, “weightlessness” [183], falling phase [182] or critical phase, during which the human body experiences a temporal weightless state provoked by a hasty movement toward the ground. The force of gravity is permanently influencing the measured acceleration. Therefore, throughout this short interval (300–500 ms) of time, the tri-axis accelerometer yields values (for the three axes) near to zero (typically lower than 0.6 g).
3. The impact or critical phase, characterized by a vertical shock. When the body hits the ground, a sudden peak of the acceleration magnitude, higher than 1.8 g [156], is measured by the accelerometer. In some cases, the initial hit can be followed by a series of minor impacts (lasting for some seconds) that can also provoke “secondary” drops and peaks of the acceleration module [183].
4. A post-fall, stability, “resting” [182] or “adjustment” [128] phase, in which the body lies on the ground. Sensor as the gyroscope can be used to recognize a remarkable change in body’s orientation that takes place after the fall.
5. A recovery phase during which the patient may remain still motionless if he/she is unconscious or severely injured after the collapse. Otherwise, ADLs can be resumed.

These phases are not present in all types of falls. Fudickar *et al.* remark in [106] that the post-fall phase may be quite different depending on the fall type. As commented, the existence of loss of consciousness clearly determines the mobility of the accelerometer after the fall. Similarly, a long fall (e.g., falling down the stairs) may be characterized by the presence of successive free fall and impact phases. Nevertheless, as a general rule, the fall analysis should be focused on more than one phase to improve the reliability of the detector. The importance for the detection of each phase is experimentally evaluated by Mehner *et al.* in [101]. In that work the results of a TBA-based algorithm seemed to improve as more phases were considered (although the experiments that took into account the free fall phase exhibited a lower performance). The same authors also concluded that the election of the time window to discriminate the post-fall phase after the impact strongly affected the detection precision. In the same line, Fudickar *et al.* develop in [106] a fall detection simulator to evaluate different TBA fall detection algorithms (one with and one without the free fall phase detection). These authors also stated that the exclusion of the free-fall detection step enhances the sensitivity, while not affecting the algorithms’ specificity. In any case, in most detection schemes, the algorithm is programmed to react if a drop in the acceleration caused by a free fall after a loss of balance is followed by an eventual and sharp increase of the acceleration. The main advantage of considering these two phases is that low freefall and high impact accelerations can be easily detected by setting simple thresholds during two consecutive observation windows [16,17]. Conversely, the possible stationary or post-fall phase (which is normally evaluated by estimating the final user’s posture or velocity) is not always analyzed. The last column in Table 2 indicates those systems where this phase is examined to confirm the fall occurrence.

**Table 2:** Analysis of the proposals: characteristics of the fall detection algorithm.

Ref.	Type of Detection Algorithm -TBA (Threshold -Based Approach)-Pattern recognition method (PRM):	Threshold (or Decision) Variable(s)	Type of Threshold: Fixed/Adaptive	Training Phase Required	Stationary or Post-Fall Phase Considered?
[6]	TBA	Low pass filtered acceleration	Fixed (based on measurements)	No	No
[53]	TBA	SMV (and position)	Fixed (user-configurable according to the phone position)	No	Yes
[55]	PRM (hierarchical rule-based algorithms to detect mobility patterns)	Acceleration components and orientation data	Fixed rules	Yes (to set the thresholds for the classification rules)	No
[61]	TBA ( mobility detection)	RMS of High Pass Filtered acceleration	Fixed	NF	NF
[69]	PRM: decision tree based on Hjorth mobility and complexity	Energy integral of the SMV and orientation data captured by the gyroscope	-	Yes	NC
[72]	TBA	SMV	Fixed	No	No
[73]	TBA	SMV	Fixed	No	No
[76]	TBA	SMV and final orientation	Fixed (based on measurements)	No	Yes
[82]	TBA	Discrete wavelet transform of the acceleration	Fixed	No	No
[94]	PRM (Mann–Whitney test to discriminate activities)	Acceleration components (plus camera data to detect activity detection)	Fixed (based on real data)	Movement patterns must be previously characterized.	No
[95]	TBA	SMV	Fixed (based on measurements)	No	No
[99]	TBA and state machine-based	SMV	Fixed (based on measurements)	No	Yes
[101]	TBA	SMV (3 thresholds for 3 phases)	Fixed (user-configurable)	No	Yes
[103]	PRM (machine learning classifiers: support vector machines, sparse multinomial logistic regression, Naïve Bayes, k-nearest neighbors, and decision trees.)	Acceleration components Classification based on a set of features extracted from the tri-axis accelerometry values (histograms, Fourier components, mean, cross products of the acceleration components, …)	No thresholds employed	Not commented	No
[105]	Combination of TBA and PRM: Tested classification algorithms: two variants of k-nearest neighbor and Support Vector Machine	SMV (for the TBA) and novelty detection techniques.	Fixed (for the TBA)	Yes	No
[110] [111]	TBA	SMV, acceleration in the absolute vertical direction, and strength of magnetic field (through Hausdorff distance) around the phone (only in [111])	Fixed	No	No
[112]	PRM: Support-Vector Machine classifiers	(Presumed) acceleration components	NC	Yes	No
[113]	TBA	SMV Orientation change after the fall above a threshold	Fixed	No	Yes
[114]	TBA	SMV	Fixed (based on measurements)	No	No
[115]	TBA	SMV	Fixed	No	Yes
[116]	TBA	SMV (during four phases)	Fixed (based on measurements)	Yes (to set the thresholds)	Yes
[118] [117]	TBA	SMV (two phases and two thresholds are considered) and orientation	Fixed (based on measurements for different positions of the phone)	No	Yes
[119]	PRM: finite state machine	Acceleration components	Fixed	No	Not considered
[120]	PRM: self-organizing map (SOM)	Waveform of the acceleration components	No thresholds employed	Yes	Yes
[121]	TBA	Acceleration components	Fixed (10g)	No	No
[122]	TBA combined with a Classification Engine that uses a neural network	SMV	Fixed (3G)	Yes	Yes
[123]	PRM: spectral comparison using reference data	FFT of the waveform captured by the Doppler sensor: average spectral ratio	Based on measurements	Yes	No
[124]	TBA	SMV and vertical acceleration	Fixed (based on measurements)	No	No
[125]	TBA	SMV (combined with the measurement of other vital signals: ECG inspection)	Adaptive (threshold depends on the user’s Body Mass Index)	No	No
[126] [127]	TBA	Three variables are considered: SMV, Signal Magnitude Area, Tilt angle.	Fixed	No	Not commented
		difference of the orientation, time between the maximum and the minimum			
[129]	TBA	SMV and angle of rotation centered on each axis	Fixed (based on measurements)	No	No
[130]	Presumed TBA	Acceleration components	Not commented	Not commented	No
[131]	Not commented	Acceleration components and orientation	Not commented	Not commented	Yes
[132]	TBA and PRM (Supervised learning)	SMV and orientation	Adaptive: thresholds are set depending on the initial position and a decision tree	Yes	No
[133]	TBA	SMV and orientation	Fixed	No	Yes
[134]	TBA: Binary Decision tree	SMV and tilting angle	Fixed	No	Yes
[136]	TBA	SMV and acceleration components	Fixed	No	Yes
[137]	NC (Detection algorithm not described)	Not commented	Not commented	No	No
[138]	TBA	Acceleration components and orientation (tilting) angle System is only focused on detecting bed falls	Fixed (angle)	No	No
[139]	TBA	SMV	Fixed	No	Yes
[140]	TBA	SMV, orientation angles (roll, pitch)	Fixed	No	No
[141]	TBA	SMV, Deviation of the accelerometry components	Not commented	No	No
[142]	TBA	SMV and rotation (computed from Roll, pitch, yaw)	Fixed	Yes (to set the thresholds)	No
[143]	Not commented (based on the accelerometry data)	NC	Not commented	Not commented	No
[144]	TBA	SWM and tilt angle	Fixed	Yes (to set the thresholds)	Yes
[145]	Presumed TBA	Tilt angle	Not commented	Not commented	No
[146]	TBA	SMV, orientation angles (roll, pitch)	Fixed(based on measurements)	Yes (to set the thresholds)	No
[147]	TBA	SMV, vertical acceleration and orientation	Fixed	No	Yes
[148]	PRM: Combined algorithm of Fisher’s discriminant ratio criterion and 𝐽3 criterion for feature selection	Statistical features derived from acceleration components, angular velocity and orientation data	No thresholds employed	Yes	No
[149]	TBA	SMV (combined with the measurement of other vital signals: ECG inspection)	Fixed	No	Yes
[150]	PRM (Supervised learning): Different algorithms for feature selection and event classification are evaluated	Mobility Pattern recognition based on a set of statistical features derived from acceleration components	No thresholds employed	Yes	No
[151]	TBA	Acceleration components (metric not specified)	Fixed	No	No
[152]	TBA	Displacement during an interval (calculated from the integration of the acceleration components)	Fixed	No	No
[153] [154]	PRM (Petri Nets and fuzzy logic)	SMV and frequency of violent vibrations	No thresholds employed	Yes (assumed)	No
[155]	TBA	SMV (during two phases: pre-fall and impact)	Fixed	No	No
[156]	Combination of TBA and PRM: State Machine, frequency component analysis (STFT Analysis, High-pass Filtering, Haar DWT, Discrete Wavelet Transform)	SMV (for the TBA), Acceleration components and orientation.	Fixed (based on a training phase)	Yes	No
[157]	PRM: State Machine, Decision Trees, K-Nearest-Neighbors (KNN) and Naïve Bayes	Acceleration components	Fixed (based on measurements)	Yes	Yes
[158]	TBA	SMV and orientation	Fixed	No	No
[159]	TBA	Acceleration components and pitch	Fixed	No	No
[160]	TBA	Cumulative sum of the Acceleration coordinates	Fixed	No	No
[161]	PRM	Nearest neighbor rule	Fixed	Yes	No
[162]	PRM	Genetic Programming	Adaptive	Yes	No
[163]	TBA	SMV and orientation data	Fixed	No	No
[164]	TBA (four algorithms compared)	SMV, Acceleration components, orientation angles (roll, pitch)	Fixed	No	Yes
[165]	TBA	SMV and variation of the position angle	Fixed (several tested)	No	Yes
[166]	PRM (Pose Body Model based on Extended Kalman filters and SVM)	Angular position, angular rate, angular acceleration. Radius curvature	No thresholds employed	Yes	Yes
[167]	PRM (Neural network: trained multilayer perceptron)	SMV and angular velocity in each axis	No thresholds employed	Yes (a database of falls and ADLs is generated)	Yes
[168]	TBA	SMV and vector angle	Fixed (based on measurements)	Yes (to set the thresholds)	Yes
[169]	TBA	SMV	Fixed	Yes (to set the thresholds)	Yes
[170]	TBA	SMV	Fixed	Yes (to set the thresholds)	Yes
[171]	TBA	SMV	Fixed	Yes (to set the thresholds)	Yes
[172]	TBA	SMV (assumed)	Fixed	No	No

## 6. Typology of the Reaction and Emitted Alarms after Detecting a Fall

An interesting aspect that is often not analyzed in detail by the states-of-the-art on fall detection schemes is the response that these systems provide once a body fall is either detected or predicted. These responses can be emitted to the monitored patient or his/her immediate surrounding environment (local response) or transmitted to one or several Remote Monitoring Users (RMUs). In this sense, the use of commercial Android devices (such as tablets and, especially, smartphones) highly facilitates both the development of local interfaces and the wireless transmission of the alarms to the RMUs.

The local response may include the automatic activation of complex assistive appliances (e.g., airbags, smart canes, smart shoes, *etc.*) to prevent the fall or alleviate its effects. Some architectures are more oriented to prevention [49], so they produce a response as soon as the patient’s gait pattern seem to indicate a potential fall. However, the local response is more commonly limited to the emission of a visual, audible and/or vibrational alarm to warn the persons who walk or stay near the patient in that moment. This local alarm can be also used as a feedback to the patient, who could deactivate the process that triggers the remote response in case that an incorrect detection (or false positive) has occurred. The second column of Table 3 specifies the type of local response that the systems in the literature offer after detecting a fall. The third column in the same Table indicates if the systems contemplate a warning period during which the patient is both notified that a fall has been detected (or predicted) and authorized to cancel the emission of the remote alarm. The interaction between the system and the patient can be enhanced by implementing a “panic button” [131,151], so that an alert is also triggered when the patient presses a particular key in the Android device.

The system may only feed-back a local response so that no remote monitoring takes place, but the majority of proposals include some mechanism to forewarn the RMUs. Due to the multimodal nature of the wireless communications of Android Smartphones, several types of technologies (Wi-Fi, 3G/4G, Bluetooth or BT) and notifications (ranging from voice calls to simple SMS to a predetermined phone number) can be programmed with just some lines of Android code to transmit the alarm. The fourth and fifth columns in Table 3 indicate which technology is employed and which typology of message is emitted in the analyzed systems. As it is clarified in the sixth column of the table, the alert message can be complemented (apart from personal preconfigured data of the monitored user) with other supplementary information such as the location (which can be easily determined via the GPS module that is incorporated in most SPs), a timestamp or even the signals of different sensors. In this situation, a smartphone could also act as a gateway for the biosignals (e.g., ECG) that are sensed by other wearable (wireless or wired) medical devices.

On the other hand, the information related to the fall events or even the measurements that are constantly performed by the system sensors can be stored by the Android platform, which would behave as a “data logger” unit. Making so, the sensed magnitudes (e.g., the circumstances preceding a fall) could be analyzed offline. Alternatively, these data can be periodically or eventually transmitted to a remote (normally Internet-connected) central data server so that the patient’s activity and parameters can be tracked online by the RMUs.

**Table 3:** Analysis of the proposals: characteristics of the fall reaction.

	Local Reaction		Remote Alarm Transmission			Logged Data	Typology of RMU
Ref.	Type of Local Alarm	User Feedback (Alarm Stop)	Transmission Technology	Type of Remote Alarm	Transmitted Data (Apart from Fall Status and User ID)	Stored Data	
[6]	Visual signal	Yes	TCP/IP socket (presumed Wi-Fi, 3G/4G)	Not commented	Not commented	Not commented	Web page
[53]	Vibration, visual alarm and audio message	Yes	Cellular telephony	SMS, phone call	Timestamp, GPS location and password	Not implemented	Cell phone
[55]	Acoustic alarm	Yes	No remote alert is sent	-	-	-	-
[61]	Acoustic Alarm	Yes	3/4 G	Multimedia flow (technology is not commented)	ECG signal. GPS location	Biosignals (SPO2, ECG signals)	Web page
[69]	Text message and vibration	No	No remote alert is emitted	-	-	-	-
[72]	Audio alarm (voice message)	Yes	No remote alert is emitted	-	-	-	-
[73]	Not commented	No	Alerting just suggested	Not commented	GPS location	Not commented	Not commented
[76]	Acoustic alarm	Yes	Cellular telephony /Wi-Fi through SSL protocol	SMS, email	Accelerometer data	Accelerometer data (in a SD card of SP)	Cell phone, email client
[82]	Acoustic alarm	Yes	(presumed) 3G/4G	SMS, email, Twitter messages	GPS location	Not commented	Cell phone, email client, web page
[94]	Not commented	No	(Presumed) 3G/4G/Wi-Fi	Visual signal in a Web page	Position, type of performed activity	Not commented	Web application
[95]	Not commented	No	BT between the sensor and the SP. 3G/Wi-Fi to the RMU	Voice call, SMS, alert message to a central server	Not commented	Not commented	Cell phone
[99]	Not commented	No	Cell telephony	SMS, email	Not commented	Not commented	Cell phone, email client
[101]	Acoustic and visual alarm	Yes	Cellular telephony	SMS	Not commented	Not commented	Cell phone
[103]	Not commented	No	No remote alert is sent	-	-	-	-
[105]	Not commented	No	Wi-Fi	No remote real-time alert is emitted	-	Acceleration data is stored in the SP and transmitted to a	Off line analysis of the recorded data in a server
						server after the monitoring period	
[110] [111]	Acoustic Alarm	No	No remote alert is sent	-	-	-	-
[112]	Not commented	Yes	3G/Wi-Fi	e-mails, SMS, pop-ups on installed computer widgets	GPS location, user’s information	Not commented	Email client, cell phone, Web application & Widget
[113]	Acoustic and visual alarm, phone vibrations	Yes	Wi-Fi/3G/4G	Multimedia flow (not specifically commented)	Timestamp and GPS location	Diverse biosignals	Web page, iPhone and Droid applications
[114]	Local sound alert	Yes	Cellular telephony	SMS	GPS location, date, time	Not commented	Cell phone
[115]	Acoustic and visual alarm	No	Cellular telephony	SMS	GPS location	Not commented	Cell phone
[116]	Acoustic Alarm	Yes	3G/4G (presumed)	SMS, email	Not commented	Not commented	Cell phone, email client
[118] [117]	Not commented	No	No remote alert is sent	-	-	-	-
[119]	Message	No	No remote alert is sent	-	-	-	-
[120]	Not commented	No	No remote alert is sent	-	-	-	-
[121]	Not commented	No	No remote alert is sent	-	-	-	-
[122]	Acoustic alarm	Yes	Cell telephony	SMS	GPS location	Not commented	Cell phone
[123]	Not commented	No	Ethernet	Not commented	Sensed data	Sensed data	External database
[124]	Visual alarm	Yes	Cellular telephony	SMS	Not commented	Timestamp (logged in the SP)	Cell phone
[125]	Visual alarm	Yes	Cellular telephony	SMS	Not commented	Not commented	Cell phone
[126] [127]	Not commented	No	Not commented	MMS	timestamp, GPS location, and Google map	Acceleration data (local SQLite database in the SP)	Cell phone
[128]	Acoustic alarm, phone vibrations, tips to the user	Yes	Cellular telephony (presumed)	Message (SMS presumed)	Timestamp, location and the personal health information	Not commented	Cell phone
[129]	Not commented	No	No remote alert is sent	-	-	Acceleration data (in the SP)	-
[130]	Not commented	No	Cellular telephony	SMS, MMS or phone call	Heart rate, body temperature, tilt and fall of the patient	Heart rate, body temperature, tilt and fall of the patient	Cell phone
[131]	Acoustic and visual alarm	Yes	Cellular telephony	SMS	GPS location	GPS data in an external database	Web page and Mobile app
[132]	Not commented	Yes	No remote alert is sent	-	-	-	-
[133]	Audio alarm	Yes	3G/4G/Wi-Fi	Email/SMS to the RMU, SSL connection to a server	Inertial signals (to the server)	Acceleration data stored in the SP and in a server	Email client, cell phone, Web page
[134]	Acceleration data are displayed on the SP	No	No remote alert is emitted	-	-	Acceleration data stored in the SP	-
[136]	Not commented	No	No remote alert is sent	-	-	-	-
[137]	Not commented	No	3G/4G	SMS, phone call	GPS location	Fall history (in a web server)	Cell phone, Web page
[138]	Visual and sound alarm	No	No remote alert is sent	-	-	-	-
[139]	Alert (type not commented)	No	3G/Wi-Fi	Not commented	Biosignals from medical sensors	Not commented	Mobile app
[140]	Acoustic alarm	Yes	Cellular telephony (presumed)	Message (presumed SMS)	Oxygen saturation values, GPS location and fall direction	Oxygen saturation values, GPS location and fall direction	Smart-home database (not described)
[141]	Phone vibrations	Yes	No remote alert is sent	-	-	Acceleration data stored in a local SD card	-
[142]	Not commented	No	3G/4G (presumed)	email	Timestamp, GPS location	Not commented	Email client
[143]	Buzzer	No	Cellular telephony	Phone call, SMS, XML file	GPS location	User status	Cell Phone
[144]	Not commented	Yes	Cellular telephony	SMS	Not commented	Not commented	Cell phone
[145]	Not commented	No	Cellular telephony	Phone call	A set of health parameters	Not commented	Cell phone
[146]	Not commented	No	No alert is sent	-	-	-	-
[147]	Not commented	No	Cellular telephony	SMS	GPS location	Not commented	Cell phone
[148]	Not commented	No	Cellular telephony	MMS	GPS location	Acceleration and gyroscope data, orientation signals stored in SP	Cell phone
[149]	Visual alarms and notifications on a biofeedback application	No	Cellular telephony/Wi-Fi	SMS and email (to RMU), message using HTTP protocol and REST Web services (to a database)	Values from the medical sensors, GPS coordinates	Values from the medical sensors, GPS data	Cell phone, email client, HTTP client
[150]	Visual alarm	Yes	Cellular telephony	SMS	GPS location	Not commented	Cell phone
[151]	Visual alarm	Yes	Wi-Fi	Email, TCP/IP socket	Accelerometer data	NC	Email client, Monitoring application in a PC
[152]	Not commented	Yes	3G	SMS	GPS location	Not commented	Cell phone
[153] [154]	Not commented	Yes	No remote alert is sent	-	-	-	-
[155]	Visual alarm Audible alarm after fall	Yes	Cellular telephony	SMS	Timestamp, GPS location or cell-tower positioning (indoors)	Not commented	Cell phone
[156]	Acoustic alarm	No	Alerting just suggested	Not commented	GPS location	Not commented	Not commented
[157]	Acoustic alarm	No	3G/4G/Wi-Fi	email, SMS	Timestamp, GPS location and a link to Google maps	Not commented	Not commented
[158]	Not commented	Yes	3G/4G/Wi-Fi	SMS	GPS location	GPS data	Android App
[159]	Not commented	Yes	3G/4G/Wi-Fi	SMS, video call	GPS location	GPS data (locally stored in the SP)	3G/4G cell phone
[160]	Small vibration of a watch	Yes	BT between the watch and the SP, 3G/4G/Wi-Fi to the RMU	Email, call or SMS (suggested)	Not commented	Not commented	Not commented
[161]	Acoustic and visual alarm	Yes	3G/4G/Wi-Fi	Call and message to a Web server	Not commented	Not commented	Web interface in a server
[162]	Not commented	No	No remote alert is sent	-	-	-	-
[163]	Google Speech recognizer is launched	Yes	Mobile telephony	Voice Call	Not commented	Not commented	Phone
[164]	Acoustic alarm	Yes	Cell telephony	SMS, phone call	GPS location	Not commented	Cell phone
[165]	Acoustic and visual alarm	Yes	Cell telephony	SMS, phone call	GPS location	Acceleration data, GPS data, date	Cell phone
[167]	Acoustic alarm	Yes	Cell telephony	SMS	GPS location	Not commented	Cell phone
[168]	Vibration and acoustic alarm	No	Cell telephony	SMS	Not commented	Not commented	Cell phone
[169]	Not commented	No	No remote alert is sent	-	-	-	-
[170]	Vibrations	No	Cell telephony	SMS	Not commented	Not commented	Cell phone
[171]	Not commented	No	3G/4G/Wi-Fi	Message to a PHP server	Unspecified User data	Not commented	Web page
[172]	Not commented	No (Feedback call from medical staff)	Cell telephony 3G/4G/Wi-Fi (assumed) to connect to a server	Message to a remote server, SMS	Accelerometry data	Acceleration data, alarms	Web page

These central servers are not always required as long as alarms can be directly delivered to the cell phone of the RMU via SMS, MMS or voice calls, so that a specific interface for the RMU is not required. A direct connection through a TCP/IP socket can also be established between the patient and a certain application running in the terminal of the RMU (e.g., a laptop). However, the use of a server usually permits more flexible and efficient access, management, processing and presentation of the information while it resolves the scalability problems if more than one RMU is required in the system. Furthermore, Web servers allow publishing the tracking information about the patient to an arbitrary number of ubiquitous RMUs. The last column of Table 3 describes the typology of the RMU of those analyzed systems that utilize database servers and/or deploy specialized applications to receive and visualize the alarms or the tracking data. In some cases, these interfaces enable the remote configuration of the FDS.

## 7. Evaluation of the Fall Detection Systems

A rigorous and systematic evaluation of fall detection schemes is essential to assess the actual applicability of these emergency systems in real-life situations. The evaluation of a wearable fall detection system must be a multidimensional task. Most studies in the literature just appraise the capability of the system to discriminate falls from other movements but a complete evaluation should contemplate other technical factors, such as robustness, system autonomy, coverage area or computing power. Moreover, the analysis should not either neglect the human aspects (ergonomics, user acceptance, *etc.*) resulting from applying this technology to the elderly.

As it is highlighted in [85], the most critical issue is to achieve a consensus in the scientific community not only about the definition of a fall but also about the protocols and procedures that must be accomplished for the evaluation of FDSs. Every work describing a new architecture proposes its own experimental procedures and testbeds to evaluate the prototype. The proposals are very rarely contrasted against previous works. Just very few studies [161,162,164,184] offer comparatives of the performance of different detection algorithms. The election of the individuals under test does not obey the same criteria while the descriptions offered by the works about the testbeds do not follow a normalized pattern. Only in [110] Dai *et al.* compare the performance of the proposed scheme with that of a commercial (not-Android) fall detector.

Although several studies have tried to establish a common evaluation benchmark, only a reduced set of aspects of the evaluation procedures are normally addressed by most works. Table 4 summarizes the basic characteristics of the evaluation experiments that are performed by the analyzed works to characterize the performance of the system. A quick look on these data reveals the heterogeneity of the evaluation tests (in fact, there is a not negligible amount of works that do not evaluate their own proposed FDS).

The table indicates if the validation experiments were performed with real life or emulated falls. Columns 3 to 8 describe in turn the number and characteristics of the experimental individuals, the position of the Android device, and the number and types of the evaluated falls and ADLs, respectively. Column 9 specifies the metrics that are measured to assess the effectiveness of the detection algorithm. Columns 10 and 11 indicate if the performance of the Android device (consumption of battery and computing resources, capability of coexistence with other running applications) is investigated. Finally, columns 12 and 13 informs about the SP model and Android version that are employed in the experiments.

The next sub-sections discuss the main issues related to these aspects of the system’s evaluation.

### 7.1. Emulation of Falls

Due to the difficulty of evaluating the detection systems by monitoring real body falls (especially in a population of old people), the vast majority of the proposals are tested by emulated falls, normally performed by a short group (1–8) of young and healthy adults (in [110,111] Dai employ mannequins for this purpose). In most cases, during the experiments the subjects under test are asked to emulate a fall (or a certain ADL) and tumble onto a pad (which may in turn introduce another important divergence in the mobility pattern when compared with that of an actual fall to the ground). Just a few studies consider mobility data from monitored elderly [144] or actual data from databases. Obviously, for the sake of safety, in the case of using elderly people as experimental subjects, they are only asked to execute ADLs. In [185] samples are obtained by making both young and elderly subjects to walk on a treadmill. Subjects are equipped with a safety harness to protect them in case of a fall.

The validity of the tests with mimicked falls has been mistrusted as far as real-life falls happen in a faster and jerkier way than imitated ones [101]. The comparison performed in [186] (based on a reduced number of falls suffered by elderly) indicates that the dynamics of simulated falls and real-world falls can be rather different. Bagalà *et al.* [48] also evidenced that the detection effectiveness decreases when facing real world falls. In this regard, authors highlight the need of testing fall-detection algorithms in real-life conditions and the importance of employing a real-world fall database during the evaluation of the FDS. Quite the opposite, the study in [187] employed acceleration data from actual falls among older people, but the results seem to show that real-life falls exhibit similar features as the intentional “laboratory” falls that are typically emulated to test the detectors.

Apart from fall emulation, another strategy for the evaluation is the long-term monitoring of the individuals under test. The system presented by Huq in [172] was evaluated by long-term monitoring of 54 elderly volunteers (although after several months of permanent tracking, just twelve falls were reported). Albert *et al.* investigate in [103] if their proposed FDS distinguishes real fall events (in a sample of eight week-long recordings) from simulated falls. The European FATE program has designed an architecture with a specific fall detection hardware [143] which was planned to be evaluated by tracing a set of patients in different countries during a period of twelve months.

In addition, it is still an open issue if the records obtained from falls emulated by healthy young people can be considered representative of older people’s actual falls [105]. In this point we have to take into account that some detection systems must be parametrized and adapted to the particular mobility pattern of a certain patient. Majumder *et al.* show in [69] that the accuracy of mobility pattern classifiers can be poor (less than 30%) when one subject’s gait is classified based on the other subject’s dynamics.

In [161] after a thorough analysis of the performance of a detection algorithm based on a nearest neighbor rule, Medrano *et al.* conclude that that the adaption of the detection algorithm to the user to be monitored is a key aspect to decrease the number of false alarms. In fact, these authors state that personalization should become an important research issue in this field as long as the target audience of fall detector system (elderly) might exhibit different characteristics from those subjects (young or middle-age volunteers) involved in the evaluation experiments of the detection applications. The study in [188] compared the acceleration data obtained from emulated falls and those monitored in a set of elderly people who were tracked during a period six-month. From the comparison authors come to the conclusion that there are correspondences between the real-life falls of older people and the falls emulated by middle-aged individuals. Nevertheless, some mobility characteristics measured in the experimental falls were not detectable in the acceleration signals of the actual scenario.

### 7.2. Typology of Falls and Activities of Daily Life (ADLs)

The effectiveness of a FDS must be contrasted against a wide variety of fall types and ADLs. Typical falls in elderly people have been classified in detail in [84]. In that paper, Yu characterizes the duration and the dynamics of the falls by considering three types: falls from sleeping (or from the bed), fall from sitting (or from a chair) and falls from standing or working. Based on that classification, Abbate *et al.* [90] propose a systematic set of 20 types of falls (and 16 different ADLs) as standard trial scenarios for FDSs.

In most works, ADLs are also typified as a set of particular situations and movements. As abovementioned, just in some cases [161] the systems are tested through real world experiments involving subjects that transport the wearable Android device during several hours or days. The characteristics of the ADLs and falls emulated by studies on fall detections systems have been thoroughly studied in the state-of-the-art presented in [64]. As it can be deduced from Table 4, research community is far from having reached a general agreement about the nature and quantity of the falls and ADLs that must be emulated to test the systems.

### 7.3. Position of the Android Device

As it could be expected, the position in which the mobility sensor is worn by the patient strongly affects the behavior of the fall detector. In some works (see Table 4) different positions are tested and compared.

The positions where the performance of a wearable system seems to yield the best results are the chest and the waist [124], probably because they are closer to the center of gravity of a human body (whose location in turn changes from males to females). In this sense, a compromise between the system efficiency and the patient’s comfort must be achieved. The main limitation of some approaches is that the system (and the detection algorithm) is designed assuming that the SP is worn in a very particular and optimized placement (and even fixed with an adjustable band or a similar garment). This makes the application of those SP-based systems quite unrealistic. In fact, the location of the detection smartphone in a very specific and “unnatural” position may impose the need of carrying an extra device (apart from the personal phone which is normally operated by the patient). This may clearly lessen the attractiveness of FDSs for their potential users [189].

When compared to the chest, waist placement ergonomically reduces the user discomfort while imposing little constraint on body movements [114]. The thesis in [187] concludes that a waist-worn fall detector with a simple threshold-based algorithm can accurately discriminate falls from ADLs. However, people normally prefer to put the smartphone in the shirt or pants pockets rather than keeping it on the waist.

The use of pockets may reduce the attachment of the phone to the body and, consequently, the capability of the sensed magnitudes to characterize the human movements. This loose attachment straightforwardly diminishes the effectiveness of the designed algorithm. Kau *et al.* showed in [156] that the detection efficiency degrades if the SP wavers within a pocket. The work in [162] evaluates the performance of both a threshold-based and a genetic programming method. The tests are repeated with two options for the SP location: tight pants pocket and loose pants pocket. Results indicate that the performance of the threshold technique deteriorates for the loose pant pocket setting, which introduces noise in the measurements of the acceleration.

The advantages of using an Android smartwatch (instead of a smartphone) to track the movements are the ease and comfort of use, the high quality of acquired sensor data, the minimum weight and the low power consumption characteristics [160]. However, the high and permanent mobility of the wrist is usually distant from being a good indicator of the body stability.

### 7.4. A Proposal for Defining Databases of ADL and Fall Mobility Samples for Evaluating Smartphone-Based Detection Systems

As previously suggested, one of the key problems in the evaluation of FDSs is the lack of a common database of realistic mobility patterns that enable a comprehensive comparison of the different algorithms proposed by the literature. The need for clinical databases of real-world fall signals has been remarked in [91]. In fact, for every new proposal, authors arbitrarily select which type of ADLs and falls are employed during the evaluation.

The Human Compute Interaction Lab of South China University of Technology offer [190] a dataset (SCUT-NAA), described in [191], consisting of different acceleration-based activity samples from 44 individuals performing 10 different ADLs. The movements were iterated by alternatively locating the wearable tri-axis accelerometer in three different positions (waist belt, trousers pocket and shirt pocket). However, the samples, which were aimed at assisting the research community in the field of activity recognition, do not include falls.

The Telecommunication System Team (TST) at the Università Politecnica delle Marche (Italy) has published an interesting fall detection database (fully described in [49] and publicly available at [192]), consisting of ADLs and fall actions emulated by 11 volunteers. The traces include two raw acceleration streams, captured not by Android devices but by specific wearable Shimmer sensors attached to the waist and right wrist of the volunteers.

Except from some studies, such as [105], most works in the literature that propose new fall detection methods do not make available the data (acceleration and gyroscope signals, *etc.*) employed to evaluate the architecture. As a consequence, the reproducibility of the results is practically unviable. Only in [48], the performance of 13 published detection algorithms is compared when they are tested with a database of mobility samples corresponding to 29 real-world falls.

Databases of samples describing fall events and ADLs should be publicly available in the Web so that researchers can share a common reference framework to compare and validate their proposals about FDSs.

There are platforms (such as Actipal [193]) which have been developed to facilitate data collection about the weight and physical activities of study participants by using a smartphone application. Nonetheless, they are generic systems that do not record the parameters that are required for a full description of a fall event.

An interesting initiative to standardize the evaluation of fall detection algorithm is promoted in [184]. That work offers a dataset of the traces of human activity emulating four types of falls and nine different ADLs. The trace is obtained by recording the data from the accelerometer and gyroscope sensors of a SP. The goal of the database is to provide a common framework to test new methods for fall detection and activity recognition,

Due to the variable nature of the experimental environment, the diversity in the mobility sensors and the ambiguous concepts of fall or ADL, public databases intended to test the accuracy of detection fall algorithms should describe in details the way in which the samples were generated. In our opinion, if Android Smartphones are planned to be employed to create a database of activity measurements, the description of the samples should contain (at least) the following information: 

1. Nature of the monitored variables. The samples captured by a smartphone should consist of a file with a sequence of records describing the temporal evolution of the movements. In most cases, every record in the file should contain a timestamp and the values of the 3-axis accelerometer coordinates (*A_x_*, *A_y_*, *A_z_*). Additionally, if the smartphone is provided with a gyroscope, every record could also incorporate the measured values of the pitch, roll and yaw attitude angles. Other variables sensed by the smartphone (such as GPS coordinates) could be also optionally incorporated to the sample files.

2. Characteristics of the employed smartphone utilized for user tracking. Each sample should clearly inform about the device (*i.e*., the smartphone model) and version of the Android OS with which the corresponding activity (fall or ADL) has been monitored. The characteristics and parameterization of the embedded sensors in the smartphone (accelerometer, gyroscope, *etc.*) should be specified. In the case of the accelerometer it is utterly necessary to report the range and, especially, the sampling rate of the device. As mentioned, this last parameter, which clearly impacts on the accuracy of the mobility traces, can be selected by the monitoring Android application by choosing one of four possible values of a certain variable. In any case, as commented, the maximum sampling rate at which the accelerometer can operate is heavily dependent on the smartphone model.

3. Origin and scenario of the analyzed movements: the database should indicate if the movements correspond to actual activities in the real life or if they are emulated by a set of controlled experiments. Similarly the scenario where the monitored movements took place should be briefly commented. For example, if movements are emulated by experimental users, it is important to indicate if cushions, mattresses or foam protection pads have been used to avoid injuries. The use of these elements could alter the captured samples as they soften the impact of the fall. Conversely, if protective pads are not employed, experimental individuals could emulate less realistic falls because of the fear of getting injured.

4. Position of the smartphone. The sample should detail the position (wrist, chest, thigh, *etc.*) where the smartphone is located for the monitoring. The log file should comment the way in which the smartphone is attached to the user body (e.g., through a specific belt, located in a pocket, *etc.*).

5. Basic characteristics of the individuals under test. These characteristics should include (at least) the gender, age, weight and physical conditions of the subjects. This description should also indicate if any of the individuals suffer from any pathology that can affect his/her mobility.

6. Typology of the monitored or emulated falls and ADLs. The database should classify and describe the positions that have been considered to simulate the falls (lateral, backwards, *etc.*). If a real fall is monitored in an actual scenario, a description of the typology should be also required. Likewise, the nature of the simulated or tracked ADLs should be characterized. Both for falls and ADLs, it would be highly recommendable to record with a video-camera the experimental users while performing their movements. Thus, if necessary, the experiments could be easily replicated and validated in another testbed. For that purpose, a file containing a video clip can be annexed to the different entries of the database with the experimental samples.

7. The code of the Android (or iOS) application that has been utilized to capture the samples should be publicly and freely available. One advantage of Android code is that it can be easily reutilized in other Android-compatible devices and openly distributed in the Internet. In spite of this, just a few of the analyzed Android detection systems (such as iFall [53] or that proposed by Kerdegari [194]) have been released. The documentation of other available apps for fall detection (Spantec Fall Detector, Fall Monitor, T3LAB, Fade Fall Detector, *etc.*) does not give any detail on the implemented detection algorithm, nor any insight into the effectiveness achieved by the software. Actually there is not any mature Android-based product in this domain (see Google Play Store [195] for the specifics of these applications).

An interesting initiative in this field is the FARSEEING (FAll Repository for the design of Smart and sElf-adaptive Environments prolonging INdependent livinG) project. The major goal of this project is to create and offer a meta-database of real-world falls. The project involved the collaboration of 40 experts from different disciplines related to fall recording and fall prevention. This panel of experts achieved a consensus in 2012 about the way a clinical dataset of falls must be configured and described. The recommendations are summarized in [196].

### 7.5. Numerical Evaluation of the Algorithms: Selection of Performance Metrics

There is not a complete consensus about the selection of the parameters that must be estimated to numerically assess the effectiveness of fall detection. In general, most papers employ metrics that are typically applied in the evaluation of systems targeted at pattern recognition and information retrieval with binary classification [197]. All these metrics are computed from four variables (*TP*, *TN*, *FP*, *FN*), where *TP* and *TN* respectively indicate the numbers of “True Positives” and “True Negatives”, that is to say, the amounts of falls and ADLs that have been correctly recognized, while *FP* and *FN* represent the numbers of “False Positive” and “False Negatives” (actual ADLs and falls that have been misidentified, respectively). Among these performance metrics we can mention the following ratios:

(2) $$Specificity\;=\frac{TN}{FP+TN}$$

(3) $$Sensitivity\;=\frac{TP}{FN+TP}$$

(4) $$Accuracy=\frac{TP+TN}{TP+FP+TN+FN}$$

(5) $$False\;Positive\;Rate\;=\frac{FP}{FP+TN}$$

(6) $$Precision\;\left(or\;Positive\;Predictive\;Value\right)=\frac{TP}{FP+TP}$$

(7) $$False\;Discovery\;Rate=1-Precision\;=\frac{FP}{FN+TN}$$

(8) $$False\;Omission\;Rate\;\left(FOR\right)\;=\frac{FN}{FN+TN}$$

(9) $$Negative\;Predictive\;Value=1-FOR\;=\frac{TN}{FN+TN}$$

(10) $$Fscore=2\frac{Precision\cdot Sensitivity}{Precision+Sensitivity}=\frac{2\cdot TP}{2\cdot TP+FN+FP}$$

In some works the exact meaning of the employed metrics is equivocal as they are ambiguously defined (*i.e.*, “percentage of false positive” or “percentage of false negatives”).

As Table 4 shows, the most popular metrics in the literature are the sensitivity (also called true positive rate, hit rate or recall) and the specificity (also called true negative rate). In Threshold-Based Approaches, these metrics are clearly determined by the detection threshold that has been set. For all TBA algorithms, a higher sensitivity is normally achieved at the cost of a lower specificity. Accordingly a trade-off between these two magnitudes must be achieved. In [105], for example, the threshold is selected to maximize the geometric mean of the specificity and sensitivity. Aiming at the same objective, there are studies that compute the Receiver Operating Characteristic (ROC) curve and analyze the evolution of the specificity and sensitivity by varying the corresponding threshold. This curve is represented by plotting the sensitivity against the False Positive Rate (FPR) when a parameter (normally the detection threshold) changes. Authors in [161] employ the area under the ROC (AUC) as the figure of merit of the detector.

The delay time of the detection system may become an important factor in those specific systems that must give an immediate response to prevent injuries. Protection devices, such as airbags, require a minimum time (e.g., 35 ms) to be effective [146]. Consequently, the delay of the detection system has also been regarded as a quality metric in very particular works. In the case of using an external sensing device, the transmission delay between the sensor and the Android platform can be also evaluated, as in [160].

### 7.6. Feasibility of Fall Detection Systems in Android Devices

The evaluation of FDSs has almost exclusively focused on the accuracy of the detection process. However, if an Android device (especially a Smartphone) is employed as a central element of the architecture, a specific assessment of the performance of the Android platform should be considered. Owing to the restricted battery capacity and computing power of most Android devices, the consumption of energy and computing resources (memory, CPU) of these devices when employed in fall detection applications must be carefully examined.

Although the consumption or the power of the proposed system is not evaluated, Zhang state in [144] that the computing resource and the extra power consumption required by the apps may become an important problem for the processor capability of the Android devices. The usual battery life of a smartphone in normal use is roughly one day [101], which can severely diminish if an additional fall detection app is being executed continuously.

The concern about battery drain of smartphones has been systematically addressed by a scarce number of studies on FDSs. In [76] authors admit that continuous monitoring in their system can endure no more than 11 hours due to battery exhaustion. Similarly, consumption tests performed in an iPhone [69] indicate that the battery consumption is really affected by the detection application (reducing the battery life to less than 3 h).

In [101] the measurements of the consumption in a Samsung Galaxy S model reveal that the detection app may require 8% of battery capacity per hour. This battery drain is almost independent of the user’s activity (*i.e.*, incidence of falls). In that study, the fall alarm just implied to send a text message without further information about the user’s status. The activation and use of GPS to send the user’s location coordinates within the alarm message can entail a relevant extra consumption [164]. The consumption provoked by the detection application that is reported in [116] decreases to 2%, 2% and 1% battery life per hour in Google Nexus One, HTC Desire and Nexus S models, respectively. Similar conclusions have been drown in [156]. This study examines the percentage of battery that is exhausted after a period of up 7 h during which the app is being permanently executed. Authors find that, after that period, the percentage on the power consumption consumed by the proposed app is around 9%, which is similar to that of an app game. Authors employ a state-machine based system. From their analysis they conclude that the classification process highly impacts on the computational costs of the system.

The consumption of SP-based fall detectors is also investigated by Mellone in [133]. Authors show that battery life strongly depends on the sampling frequency and the number of sensors that are employed for the detection. For example, in a Samsung Galaxy S II equipped with a standard battery and sampling at 100 Hz, the battery life may span from 16 h up to 30 h, if all the three sensors (magnetometer, gyroscope and accelerometer) or just one sensor (accelerometer) are utilized. In [164] authors show with systematic tests that the permanent use of the GPS sensor (to locate the patient in case of an alarm) heavily degrades the SP autonomy.

In [134], a battery lifetime of only 7 h is measured in a HTC SP when the mobile is utilized to receive the data via Bluetooth from an external accelerometer and decide if a fall has occurred.

The impact on the battery drain of the detection app in an Android-based system is evaluated in [110] too. Tests are conducted during 6 h with and without the detection app. Results show an important reduction of 10% of the battery level when the app is running. A similar analysis is undertaken in [159]. This thesis evaluates the time required to reduce the battery level of the SP from 100% to 80% with and without the fall detection app. From the results authors extrapolate that the app may reduce the lifetime of the battery by 20%.

The thesis by Viet in [198] investigates the consumption of a SP which is utilized in a mobility recognition system. The study analyses the effect of the acceleration sample frequency on the consumption revealing that a trade-off between the detector accuracy and the battery saving must be achieved. The same author proposes in [199] a forward fall detecting algorithm which is designed to lessen energy consumption without sacrificing accuracy.

Kerdegari *et al.* show in [167] that the FDS (based on a neural network) provokes up to 30% of the total battery consumption in a HTC smartphone model.

Authors in [160] also conclude that using the lowest possible accelerator sampling rate is a key aspect to minimize consumption. The smartwatch used in the system that they propose is proved to be capable of continuous sampling for at least 30 h.

In the architecture described by Aguiar *et al.* in [157], the Android app only collects the tri-axis accelerometer coordinates continuously, despite the interest of other inertial and position sensors data for fall detection. Authors justify this selection by stating that the accelerometer is the most optimized sensor as it concerns the battery usage. Another technique to moderate the battery drain (proposed in the same paper) is to downgrade the accelerometer sampling frequency to 4 Hz when the user is detected to be motionless. In any other condition the sampling frequency is configured to be 67 Hz. Making so, the authors measured that the detection app just provokes a battery consumption of 0.19% of the total capacity per hour (with the minimum sampling rate) while the consumptions increases to 2.21% per hour with the maximum sampling rate. Thus, authors conclude that the phone battery can be drastically extended with a smart management of the sensor frequency.

Finally, the work in [147] summarizes different proposals to reduce the consumption in a fall detection app: (1) to run the monitoring daemon in the background while other components of the program halt; (2) to tune the sampling frequency; (3) to launch the pattern matching only after the collected data exceeds a certain preset threshold; (4) to minimize as much as possible the activation of hardware components (especially the screen).

In any case, battery lifetimes of just one or two days may not be acceptable for some users and some types of FDSs. The experiments developed in [144] also include a questionnaire that had to be fulfilled by the users after a real life trial of a FDS combining BWS and CAS techniques. The users’ answers indicated that the system should be operative at least for half year with no battery recharge in order to be judged as satisfactory.

On the other hand, as the column 10 of Table 4 clearly illustrates, the processing (CPU) and memory resources that detection applications demand are investigated even in a smaller amount of studies than power consumption. In fact, the CPU load introduced by the detection app is only evaluated in [69,134]. In this last paper, Hou concludes that processor speed is not proved to be a remarkable system constraint [134]. In contrast, tests show that typical multitask performance of Android platforms is prone to failures that should be taken into account to evaluate the robustness of the FDSs. In this sense, the problems related to this multitask capability of Android are completely neglected by the literature. To date no study has investigated if the coexistence of a running fall detection application may affect the conventional functionalities of a smartphone (phone calling, chatting, messaging, web browsing, *etc.*). In this regard the impact of other high resource consuming apps (e.g., those for multimedia decoding) on the performance of fall detectors should be also thoroughly analyzed.

Finally, ergonomics is another essential feature that is not normally considered by the literature. The acceptability, potentials and usability of Smartphone-based wearable fall prevention systems have just been studied by a reduced number of semi-structured interviews in [200] and questionnaires in [4,122,128]. Results seem to indicate a positive reception by the users but further studies with a larger population are required for a more precise validation.

**Table 4:** Reported characteristics of the evaluation tests of the proposals in the literature (NC = not commented in the work).

Ref.	Real Life/Emulated Movements	Number of Individuals Under test	Characteristics of the Individuals (Age, Weight, *etc.*)	Tested Positions of the Android Device	No. of Iterated Falls	Type of Tested or Emulated Falls	Type of Emulated ADLs	Performance Metrics	Evaluation of Battery or Computing Consumption	Coexistence Analysis	Used Smartphone Model(s)	Version of Android
[53]	Not evaluated	-	-	-	-	-	-	-	-	-	HTC G1	-
[55]	Emulated	6	5 males, 1 female 20–52 years	Waist	144[123]	Falls ending with lying and sit-tilted	Walk, stand, run, jump, sit	Sensitivity, Specificity	Not included	No	HTC Wildfire S A510e	Android version 2.3.3.
[61]	NC	NC	NC	NC	NC	NC	NC	NC	Not included	No	NC	Android 2x
[69]	Emulated	5	20–30 years 158–169 cm 60–80 kg	User’s pocket	NC	Abnormal gaits: simulated peg leg, simulated leg length discrepancy	Walk	Accuracy (to distinguish mobility patterns) Specificity Sensitivity	Consumption	No	iPhone	-
[72]	Not evaluated	-	-	-	-	-	-	-	-	-	-	-
[73]	Not evaluated	-	-	-	-	-	-	-	-	-	NC	NC
[76]	Emulated	3	24–26 years 164–175 cm 60–66 kg	Waist belt:	67	Forwards (from two positions) backwards, lateral, falling out the bed, slide against a wall	NC	Sensitivity, Specificity	Not included	No	HTC Desire,	No
[6]	Emulated	1	Height (164 cm)	NC	100	NC	Lying	FP, FN, TP, TN	Not included	No	Pantech IM-A690S	2.3.3 (GingerBread)
[82]	Emulated	5	NC	Pocket	100	NC	Walk, sit, jump, lie	Precision, Recall	Not included	No	Nexus One	Android 2.0
[94]	Emulated in a realistic scenario (retirement home)	NC	NC	Chest	NC	NC	Walk, run, sit	Accuracy, Sensitivity, F-score	Not included	No	NC	NC
[95]	Not evaluated	-	-	-	-	-	-	-	-	-	NC	NC
[99]	Emulated	10	Young, male 26.2 ± 3 years 177 ± 5 cm 78.5 ± 5.3 kg	Pocket at the thigh position	600 (including ADL)	Some mentioned but not systematically tested	Some mentioned but not systematically tested	Sensitivity, Specificity	Not included	No	HTC Desire HD	NC
[101]	Emulated	3	NC	Waist (trouser pocket)	120	Forwards, backwards, sideway.	Walk, run, stairs walk, sit.	Sensitivity, Specificity	Consumption	No	Samsung Galaxy S Sony Xperia Ray	NC
[103]	Emulatedand Real life	15 (emulated) 9 (for 10 days)	8males/7females Aged 22–50	Waist (belt, placed on the back)	221	Left and right lateral, forwardtrips, and backward slips	NC	Sensitivity (in the detection of fall type)	Not included	No	Tmobile G1	Android OS 1.6
[105]	Emulated	10	3 Males, 7 females 20–42 years 161–184 cm 54–98 kg	Left and right pocket	48 per subject	Forwards, left and right-lateral backwards, syncope, sit on empty chair, falls with strategies to prevent the impact and falls with contact to an obstacle	Participants carried a smartphone in their pocket or hand bags for at least one week	Sensitivity, Specificity (and their geometric mean)	Not included	No	Samsung Galaxy Mini	2.2
[110] [111]	Emulated with both mannequins and real individuals	15	13 males, 2 females 20–30 years 161–190 cm 51–80 kg	Chest, waist, thigh	600 (with a mannequin)600 (humans)	Forwards, backwards, lateral	Walk, jogging, stand, sit	Sensitivity, Specificity	Consumption CPU usage	No	HTC G1	Android 1.6
[112]	Not evaluated	-	-	-	-	-	-	-	-	-	-	-
[113]	Real life patients but fall detection not evaluated		Only commented race and mean age	NC	NC	NC	NC	-	-	-	Motorola Droid Smartphone	NC
[114]	Emulated	18	12 males, 6 females29 ± 8.7 years	Waist	216 (3 per individual and type of fall)	Forwards, backwards, lateral left and lateral right	Sit-to-stand, stand-to-sit, level walk, stairs walk answer the phone, pick up an object and get up from supine	Sensitivity, Specificity	Not included	No	Google G1	NC
[115]	-	-	-	-	-	-	-	-	-	-	NC	NC
[116]	Emulated	10	26.2 ± 3.04 years, 177.6 ± 5.2 cm, 78.3 ± 5.3 kg	Thigh (trouser pocket)	600 (including ADLs)	NC	NC	Sensitivity, Specificity	Not included	No	HTC G1-Desire &HD, Samsung i7500, Google Nexus One	NC
[118] [117]	Emulated	5	22–30 years 160–175 cm	Hand, chest and pants pocket	45	Forwards, backwards, and aside	Sit, stand, walk, run, stair walk	TP, TN, FP, FN, Sensitivity, Specificity	Not included	No	Nexus One	NC
[119]	Emulated	3	Not commented	NC	5 per person (p.p.)	Forwards, backwards, and lateral falls	Walk, sit, squat, stair walk	Sensitivity and specificity	Not included	No	NC	NC
[120]	Emulated	NC	NC	NC	2 sets of 10 and 12 falls	Fall on the floor, different types of fall on an armchair	Jogging, normal walk, stairs walk, stand-sit-stand, fast walk	Analysis focused on detecting the movement type	Not included	No	NC	NC
[121]	Emulated	1	1 male, 33 years	Pocket of the shirt	NC	NC	Lying	NC	Not included	No	Samsung Galaxy SIII	Android version 4.0.4.
[122]	Database and emulated	7	20–60 years 165–177 cm 56–95 kg	Waist	44 (42 ADLs)	NC	Sit, lie, jump, run, walk, hit the sensor	Accuracy, Specificity	Not included	No	HTC Google Nexus One	NC
[123]	Emulated	4	NC	On a wall	50	NC	Walk, shake/ raise/move hands, sit, stand up, empty room	Precision, FP rate	Lined-powerNC	Specific device	BeagleBoard-XM	Android version 2.2.
[124]	Emulated	4	NC	Chest (pocket) waist, and thigh.	30 per individual	NC	Walk, sit, stand up and others (not commented)	Sensitivity, Specificity	Not included	No	HTC Desire and Tattoo	Android 1.6
[125]	Emulated	20	12 males, 8 females BMI in [20, 30.^12^] 20–50 years	Chest (shirt pocket)	400 falls and 800 ADLs	NC	Walk, sit down, jumping	FP, FN, Sensitivity, Specificity	Not included	No	HTC A3366	Android 2.2
[126] [127]	Not evaluated	-	-	-	-	-	-	-	-	-	Lenovo Le-Phone	NC
[128]	Emulated	4	3 males, 1 female 20–26	Right thigh pocket, held in hand.	100	Fall on hands, knees, the back, the left and right side of the body	Answer the phone, put the phone into the thigh pocket, sit, drop the phone from hand, walk	Precision, Recall	Not included	No	Google Nexus S	Android 2.2
[129]	Emulated	5	Several males Average age: 25.5 173 ± 5.3 cm	Waist	40 per subject	Forwards, backwards, left, and right	Walk, jogging, sit down, stand up, stairs walk	Sensitivity	Not included	No	Samsung SHW-M110S,	Android 2.3.3
[130]	Not evaluated(Evaluation limited to set the thresholds)	No	-	-	-	Dropping the accelerometer from different heights	-	-	Not included	No	NC	NC
[131]	Not evaluated	-	-	-	-	-	-	-	-	-	NC	NC
[132]	Emulated	NC	155–170 cm50–75 kg	Chest/pants pocket, hands (while talking or tapping the phone)	224	Forwards, backwards, aside bed fall (from 5 different initial positions)	Jogging, sit, stand, jump	Sensitivity, Specificity, TP, TN, FP, FN	Not included	No	Pantech Sky VegaRace	Gingerbread 2.3.3
[133]	No evaluated	-	-	-	-	-	-	SP Battery Lifetime	Consumption	No		
[134]	Emulated	10	25–35 years	Indifferent (acceleration sensor on the chest)	NC	Forwards, backwards and lateral directions	NC	Ratio between TP and total number of activities Response time	Battery lifetime, Required Memory	No	HTC	2.3
[136]	Emulated	NC	NC	NC	33	NC	NC	TP	Not included	No	NC	NC
[137]	Emulated	NC	NC	NC	NC	NC	NC	NC	Not included	No	Samsung Galaxy S III	NC
[138]	Emulated	1	NC	NC	NC	NC	Sit up, get out of bed	NC	Not included	No	NC	NC
[139]	Emulated	3	NC	NC	114	NC	No ADL tested	Sensitivity	Not included	No	NC	4.2 Jelly Bean
[140]	Real life (ice-skaters)	7	NC	Waist	50	NC (210 min of ice-skating)	Ice-skating Movements	Sensitivity, Specificity, Accuracy	Not included	No	NC	NC
[141]	Emulated in a treadmill	6	20–22 years 3 males: 171.5 cm, 66.2 kg, 3 females: 161.4 cm, 49.6 kg	Wristwatch	240	Front, back, left, and right falls	Series of 50 s moving and static	Sensitivity	Not included	No	WIMM Android watch	NC
[142]	Emulated	1	159 cm	Chest (left pocket)	NC	Backwards	Sit down quickly on a chair, lie on bed	Sensitivity Specificity Average time to complete the detection	Not included	No	HTC One S	NC
[143]	Real life (prototype aimed to be tested with actual patients)	NC	NC	NC	NC	NC	NC	NC	Not included	No	Samsung Galaxy Mini S5570	2.3.4 or higher
[144]	Emulated And real life	Emulated: 3 (training phase) 10 (test phase) Real life: 11	Test phase: Averages:24 years old, 173 cm 73 kg, Real life: 3 males, 4 females	Waist	Training: 5 falls , 7 ADLs Real life: No falls	“Simplex” and “complex” into a chair, or falls with grasping the wall	Walk , run, stairs walk, sit down,squat, rise	Emulated: Sensitivity, Specificity, Real-life: Only FP	Not included	No	HTC G3 Smartphone	2.1
[145]	Not evaluated	-	-	-	-	-	-	-	-	-	NC	NC
[146]	Emulated	30 (for test)12 (for training)	24.3 ± 2.04 years, 169 ± 4 cm, 63.17 ± 7.37 kg	Chest	NC	Unexpected slips and trip falls,	Seated, sit to stand, squat, squat to stand, get on bed, get up from bed, stairs walk, jogging	Sensitivity, Specificity, Lead time	Not included	No	Samsung Galaxy S2i9100	2.3.3
[147]	Emulated	20	24–37	Waist	800 (40 per subject)	Pushed down, slip, forwards, backwards, aside, from the chair	lie down, get up from the bed, sit on chair, get up from the chair, walk, run, stairs walk	Sensitivity,Specificity	Not included	No	NC	NC
[148]	Emulated	10	6 males, 25 ± 5 years, BMI: 23.2 ± 2.7 kg/m^2^, 4 females: 23 ± 3 years, BMI: 21.5 ± 2.2 kg/m^2^	Chest (in a band)	200 falls and ADLs	Forwards, right-side, backwards, left-side	Sit, lie, stand, Lie-to-sit, sit-to-stand, stand-to-sit, walk, stairs walk run, jump	Confusion matrix of the pattern recognition	Not included	No	Samsung I9023 Nexus S	2.3.6
[149]	Emulated	3	NC	NC	50	NC	Walk, run, sit, jump	FP, FN. TP, TN of the different mobility patterns	Not included	No	NC	NC
[150]	Emulated	8	NC	Pocket	127	Forwards, backwards, side fall, hard falls, soft falls	Stand, walk, run, stairs walk, travel in a car, brake in a car, drop the phone	Recall, Precision	Not included	No	NC	NC
[151]	Not evaluated	-	-	-	-	-	-	-	-	-	NC	NC
[152]	Not evaluated	-	-	-	-	-	-	-	-	-	NC	NC
[153] [154]	Emulated	12	Males and females 20–56 years 155–183 cm 44–72 kg	Pocket (assumed)	20 (52 ADLs)	Forwards, backwards, vertical and sideway	Walk, jogging, jump, sit down, squat down	TP, TN, FP, FN	Not included	No	NC	Android 2. 3
[155]	Emulated	8	Male. 23 ± 3.45 years 60 ± 7.68 kg	Waist	6 per subject	Lateral, back-forward fall	Sit down, stand up, walk and turn around, lie down and get up	Sensitivity, Specificity	Not included	No	NC	NC
[156]	Emulated	5 for the training phase and 5 for the tests	NC	Pocket	50	Not specified	Run, walk,sit down, stairs walk, tread, jump and wave the SP	Sensitivity,Specificity	Battery consumption	-	Sony Xperia U-series	2.3.7
[157]	Emulated	28 for the training phase and 8 for the tests	Training: 24 males, 4 females, 22–28 years, 166–184 cm, 59–83 kg. Tests: 4 males, 8 females, 63–69 years, 151–171 cm, 62–82 kg.	Pocket in a vertical positionand/or hold to the belt (centrally or laterally).	1879	10 types: backwards, forwards and lateral with diverse different ending position	1671 ADLs 4 types of recoveries from a fall, walk, lying, sitting, bending down, coughing and sneezing,	Sensitivity, Specificity, Accuracy	Percentage of battery drain per hour	-	Samsung GalaxyNexus	NC
[158]	Not evaluated	-	-	-	-	-	-	-	-	-	NC	NC
[159]	Not evaluated	-	-	-	-	-	-	-	Time to reduce the battery level from 100% to 80%	-	HTC one X, Samsung Galaxy Tab 10.1.	NC
[160]	Not specified	NC	NC	NC	NC	NC	NC	Number of false alarms and delay	NC	No	NC	NC
[161]	Emulated	10 for an offline evaluation, 4 to evaluate the system during several days of real operation	7 males, 3 women 20–42 years old 54–98 kg, 161–184 cm	NC	8000 ADL and 500 falls	Forwards, backwards, left and right lateralfalls, syncope, sit on empty chair, falls with an obstacle and falls with compensation strategies	NC	AUC (Area under the ROC curve) False positive per days	NC	No	SamsungGalaxy S II/S IV and Mini, HTC Wild Fire	NC
[162]	Emulated	1	Male in his 20 s	Loose front pant pocket, tight front pant pocket	20 (for training) 20 (for testing)	Fall while standing, while walking and from the chair	Walk, run	Accuracy, Precision, Negative Predictive Value	NC	No	Samsung Galaxy S4 GT-I9505 HTC One	NC
[163]	Emulated	1	1 young and healthy person	Attached to the chest	240	NC	Lay down	Sensitivity, Specificity,	NC	No	Samsung Galaxy S3 I9300	NC
[164]	Emulated	15	6 Male, 9 Females 15–68 years 150–190 cm Average 70 kg	Waist, pants pocket	375	Forwards, lateral and backwards	Jogging, walk, stand up, sit, answer the phone	Percentages of false positives and false negatives, ROC curve	Consumption (after 6 hours operating)	No	HTC Desire X, HTC sensation XE	Android 2.3.4
[165]	Emulated	NC	NC	Waist (external sensor in ankle)	111 (including ADLs)	NC	Sit, walk, stairs walk	TP, TN, FP, FN Sensitivity, Specificity, Accuracy	Just the consumption in external sensor assessed	No	NC	NC
[167]	Emulated	4 (tests)50 (training phase)	1 Male, 3 Females 28–40 years 160–164 cm 58–69 kg	Belt, shirt pocket, pants pocket	45 (tests) 1000 (falls and ADLs during training)	Training phase: Forwards, backwards (4 types), side left (4 types), arbitrary.	Training phase: Run, walk, jump, sit down (4 types), lie down, stand up	Sensitivity, Specificity, Accuracy	Consumption	No	HTC Incredible S, Samsung Galaxy	NC
[168]	Emulated	NC	NC	Front right area of the body	40	Forwards, backwards, left, and right	Stand up, sit, walk, stairs walk, jump, run	Sensitivity, Specificity	Not included	No	LG Optimus L9 P768	Android 4.0.4
[169]	Just evaluated to set the thresholds	-	-	-	-	-	-	-	-	-	NC	NC
[170]	Just evaluated to set the thresholds	-	-	-	-	-	-	-	-	-	Samsung Galaxy Nexus	Android 4.0
[171]	Emulated	NC	NC	Pocket	100	Fall when running, walking, jumping, standing	Sit, walk, stairs walk	Sensitivity, Specificity, Accuracy	Not included	No	Not specified	Android 4.2.1
[172]	Real	54	Elderly participants	Waist	6 actual falls	-	ADLs from users monitored during several months	Sensitivity, Specificity, false positive rate	Not included	No	Samsung i555	Android 2.2

## 8. Conclusions

This work has presented an exhaustive analysis of the challenges related to the application of Android-based technology to fall detection. To the best of our knowledge, there is not any research survey exclusively dedicated to those FDSs that utilize or are implemented on Android devices. For that purpose we have thoroughly revised the existing literature, categorizing the proposed architectures as a function of different criteria: system “wearability”, role of the Android device, employed sensors, typology of the detection algorithms, or the response in case of fall detection.

The widespread extension, programmability, built-in mobility sensors and reducing costs of Android devices (especially smartphones) make them a very appealing solution for the rapid and cost-effective deployment of non-intrusive personal fall detectors. However, in spite of the remarkable (an increasing) number of proposals, the bibliographic analysis reveals that there are a non-negligible number of still open issues that clearly reduce the attractiveness and maturity of this technology. Previous studies have drawn attention to the presumably low accuracy and range of the sensors that are embedded in existing Android devices, which could not be sufficient for an accurate fall detection decision. Android partially allows setting the sampling frequency of the built-in sensors, but the absolute value of this frequency (which also depends on the employed hardware) is not commented in most papers in the literature. The convenient position of the Android devices (when used as sensors in wearable systems) is also a key aspect to determine the effectiveness of the system. The trade-off between the complexity, accuracy and need of adjustability of the detection algorithm is another important topic: pattern recognition methods may entail high computational costs as well as long training phases in order to be tuned to the particularities (weight, mobility) of the user to be monitored. Conversely, the parameterization of simpler threshold-based algorithms, which are normally based on heuristics (or on a very rudimentary study of a few fall samples), may yield too inaccurate results.

In any case, our analysis has shown that the research community is far from having achieved a consensus about the scientific procedures that must be employed to assess the efficacy of the detectors. The heterogeneous results and conclusions obtained in the literature indicate that any fall detection architecture must be evaluated through an exhaustive test-plan with a multiplicity of movement patterns and fall situations. In fact, in very few papers the experiments of other authors are repeated or the results of the proposed algorithm are contrasted with those obtained with other proposals.

The lack of a common framework to evaluate the FDSs is evidenced in some important aspects such as the absence of a reference database of pre-recorded mobility samples (emulated or real) to compare the performance of the algorithms, the huge variability of the methodology to generate the samples (typology of the falls and ADLs, characteristics of the experimental subjects) or the inexistence of performance metrics that are universally accepted. In fact, the way in which these metrics must be evaluated is not clear either. For example, a value for the specificity higher than 0.95 (*i.e.*, 95% of actual ADLs are correctly identified) is normally reported to be an indicator of an accurate performance of the fall detector. However, this roughly implies that one out of every twenty “actions of the daily life” (e.g., sitting, walking stairs, *etc.*) will produce a “false positive”, that is to say, a false alarm. Consequently, depending on the mobility of the patient, a false alarm can take place very recurrently. We have to take into account that, in order to avoid the triggering of emergency systems, false alarms have to be manually deactivated by the monitored user (who may feel irritated if this operation occurs very often). Therefore, a specificity of 0.95 can be unacceptable for many potential users of the fall detector. In this regard, FDSs should be also evaluated in a long term basis by monitoring the test subjects during days or weeks. Metrics such as the long term rate of false alarms (or average number of false positives per day) should be estimated to assess the actual applicability of the systems, as it has been performed in the recent study by Kangas *et al.* in [201].

As pointed out, a common framework for the validation of FDSs should be established. However, a full standardization of these technological solutions for fall detection should go beyond the evaluation and cover other key issues. Among these components to be standardize we can suggest: (1) the number and typology of movement sensors; (2) the required resolution and range of the sensors (and the way their parameters are configured); (3) the location of the wearable devices and the manner they are attached to the human body; (4) the format of the sensor signals to be processed or stored; (5) the communication protocol for the exchange of information between the elements in the architecture (basically, the wearable device and the remote reception point); (7) the audiovisual interfaces with both the patient and the remote monitoring points; (8) the response in case of an alarm (actions to be undertaken after a fall occurrence); (9) the global feedback and interaction of the patient with the system; (10) the formalization of training-phases (if needed) with actual target users.

In spite of the fact that we are witnessing a remarkable expansion of medical devices employing wireless communications, standardization is still a controversial issue in the field of wireless medical sensors and e-Health applications in general. As a matter of fact, many manufacturers develop their products based on their own (proprietary) standards. In the case of wireless wearable fall detectors, the lack of a standardized protocol that addresses aspects such as data security, interoperability, *etc.* poses a significant barrier to their implantation for clinical purposes. In this regard, new proposals should consider the use of communication standards for health informatics (such as ISO/IEEE 11073).

In any case, it is not always clear which regulation must be applied to FDSs, which do not intend to substitute the functionalities of any other existing and standardized medical equipment. For example, in the USA, medical devices are categorized into three types, depending on risk. Fall detectors might be considered “lowest-risk” devices, so that they could be marketed without prior FDA (Food and Drug Administration) permission. In the case of smartphone-only based fall detectors, the detection system may consist in a simple app to be installed in the patient’s phone. The FDA guidance on mobile medical applications just regulates those apps that may compromise the patient safety if they do not operate as expected [202]. In fact, as aforementioned, diverse (unregulated) Android apps for fall detection can be currently downloaded from Google Play Store.

In addition, from the examination of the literature we can also conclude that the particular problems derived from implementing fall detection applications in an Android device are normally neglected. Although most Android devices may pose severe constraints to the battery drain, the memory or the computing capacities that a constantly running application may demand, only a few studies have investigate the effect of a fall detection monitoring app on the battery consumption. Battery lifetime may strongly affect the feasibility of this type of systems in an actual scenario where the user has to be under supervision permanently. Even rarer are those papers that also analyze the computing or memory resources that these apps require. In addition, we have to consider that Android smartphones are multi-purpose devices with different functionalities. The designer of fall detecting apps must also methodically evaluate the capacity of their own program to coexist (without interference) with other ongoing applications and services (phone calls, execution of multimedia clips, browsers, GPS-navigation tools, *etc.*).

Finally, from the review of the literature we can also remark that the design of most proposed systems is solely centered on the technical optimization of the fall detection algorithms while other social aspects are disregarded. If we consider that elderly population is the main target of these systems, usability and ergonomics should be carefully investigated. Similarly, the opinion of older users must be taken into account in the design of fall detectors.

## References

[1] Alexander B.H., Rivara F.P., Wolf M.E. (1992). The Cost and Frequency of Hospitalization for Fall-Related Injuries in Older Adults. Am. J. Public Health.

[2] Carroll N.V., Slattum P.W., Cox F.M. (2005). The Cost of Falls among the Community-Dwelling Elderly. J. Manag. Care Pharm..

[3] World Health Organization (2008). Ageing, Life Course Uni. WHO Global Report on Falls Prevention in Older Age.

[4] Yoshida S. (2007). A Global Report on Falls Prevention Epidemiology of Falls.

[5] Rothschild J.M., Bates D.W., Leape L.L. (2000). Preventable Medical Injuries in Older Patients. Arch. Intern. Med..

[6] Kwan M.M., Close J.C., Wong A.K.W., Lord S.R. (2011). Falls Incidence, Risk Factors, and Consequences in Chinese Older People: A Systematic Review. J. Am. Geriatr. Soc..

[7] Web–Based Injury Statistics Query and Reporting System (WISQARS). http://www.cdc.gov/injury/wisqars/.

[8] Injuries in the European Union, Report on Injury Statistics 2008–2010. http://ec.europa.eu/health/data_collection/docs/idb_report_2013_en.pdf.

[9] Global Burden of Diseases, Injuries, and Risk Factors Study (GBD). http://www.healthdata.org/gbd.

[10] World Health Organization (2012). Falls. Fact Sheet N 344.

[11] West J., Hippisley-Cox J., Coupland C.A., Price G., Groom L., Kendrick D., Webber E. (2004). Do Rates of Hospital Admission for Falls and Hip Fracture in Elderly People Vary by Socio-Economic Status?. Public Health.

[12] Hu G., Baker S.P. (2012). An Explanation for the Recent Increase in the Fall Death Rate among Older Americans: A Subgroup Analysis. Public Health Rep..

[13] Measuring Prevalence and Risk Factors for Fall-Related Injury in Older Adults in Low- and Middle-Income Countries: Results from the WHO Study on Global AGEing and Adult Health (SAGE). http://www.who.int/healthinfo/sage/SAGEWorkingPaper6_Wave1Falls.pdf?ua=1.

[14] Masud T., Morris R.O. (2001). Epidemiology of Falls. Age Ageing.

[15] Howland J., Lachman M.E., Peterson E.W., Cote J., Kasten L., Jette A. (1998). Covariates of Fear of Falling and Associated Activity Curtailment. Gerontologist.

[16] Stevens J.A., Corso P.S., Finkelstein E.A., Miller T.R. (2006). The Costs of Fatal and Non-Fatal Falls among Older Adults. Inj. Prev..

[17] Costs of Falls among Older Adults. http://www.cdc.gov/homeandrecreationalsafety/falls/fallcost.html.

[18] Noury N., Fleury A., Rumeau P., Bourke A., Laighin G., Rialle V., Lundy J. Fall Detection-Principles and Methods. Proceedings of the 29th Annual International Conference of the IEEE Engineering in Medicine and Biology Society (EMBS 2007).

[19] Wild D., Nayak U.S., Isaacs B. (1981). How Dangerous are Falls in Old People at Home?. Br. Med. J..

[20] The Smartphone Generation Gap Over 55? There’s no App for that. http://www2.deloitte.com/content/dam/Deloitte/global/Documents/Technology-Media-Telecommunications/gx-tmt-2014prediction-smartphone.pdf.

[21] Mobile Consumer 2014: The UK Cut, Revolution and Evolution. http://www.deloitte.co.uk/mobileuk/.

[22] Smith A. (2014). Older Adults and Technology Use.

[23] Anderson M. (2015). For Vast Majority of Seniors Who Own One, a Smartphone Equals ‘Freedom’.

[24] Mohadisdudis H.M., Ali N.M. A Study of Smartphone Usage and Barriers among the Elderly. Proceedings of the 3rd International Conference on User Science and Engineering (i-USEr).

[25] Chen K., Chan A.H., Tsang S.N. Usage of Mobile Phones amongst Elderly People in Hong Kong. Proceedings of the International Multi-Conference of Engineers and Computer Scientists (IMECS 2013).

[26] Kubik S. (2009). Motivations for Cell Phone use by Older Americans. Gerontechnology.

[27] Zhou J., Rau P.P., Salvendy G. (2014). Older Adults’ use of Smart Phones: An Investigation of the Factors Influencing the Acceptance of New Functions. Behav. Inf. Technol..

[28] Plaza I., MartíN L., Martin S., Medrano C. (2011). Mobile Applications in an Aging Society: Status and Trends. J. Syst. Softw..

[29] Igual R., Medrano C., Plaza I. (2013). Challenges, Issues and Trends in Fall Detection Systems. Biomed. Eng. Online.

[30] Melander-Wikman A., Fältholm Y., Gard G. (2008). Safety *vs*. Privacy: Elderly Persons’ Experiences of a Mobile Safety Alarm. Health Soc. Care Community.

[31] Mubashir M., Shao L., Seed L. (2013). A Survey on Fall Detection: Principles and Approaches. Neurocomputing.

[32] Cheffena M. (2015). Fall Detection using Smartphone Audio Features. IEEE J. Biomed. Health Inform..

[33] Anderson D., Luke R.H., Keller J.M., Skubic M., Rantz M., Aud M. (2009). Linguistic Summarization of Video for Fall Detection Using Voxel Person and Fuzzy Logic. Comput. Vis. Image Underst..

[34] Cucchiara R., Prati A., Vezzani R. (2007). A Multi-Camera Vision System for Fall Detection and Alarm Generation. Expert Syst..

[35] Diraco G., Leone A., Siciliano P. An Active Vision System for Fall Detection and Posture Recognition in Elderly Healthcare. Proceedings of IEEE Design, Automation & Test in Europe Conference & Exhibition (DATE 2010).

[36] Foroughi H., Naseri A., Saberi A., Yazdi H.S. An Eigenspace-Based Approach for Human Fall Detection using Integrated Time Motion Image and Neural Network. Proceedings of the 9th International Conference on Signal Processing (ICSP 2008).

[37] Fu Z., Culurciello E., Lichtsteiner P., Delbruck T. Dementia Patient Movement Detection and Fall Detection Using an Address-Event Temporal Contrast Vision Sensor. Proceedings of the IEEE International Symposium on Circuits and Systems (ISCAS 2008).

[38] Hazelhoff L., Han J. Video-Based Fall Detection in the Home Using Principal Component Analysis. Proceedings of the 10th Internationa Conference on Advanced Concepts for Intelligent Vision Systems (ACIVS 2008).

[39] Jansen B., Deklerck R. Context Aware Inactivity Recognition for Visual Fall Detection. Proceedings of the 1st International Conference on Pervasive Computing Technologies for Healthcare.

[40] Lee T., Mihailidis A. (2005). An Intelligent Emergency Response System: Preliminary Development and Testing of Automated Fall Detection. J. Telemed. Telecare.

[41] Liu C., Lee C., Lin P. (2010). A Fall Detection System using k-Nearest Neighbor Classifier. Expert Syst. Appl..

[42] Miaou S., Sung P., Huang C. Customized Human Fall Detection System using Omni-Camera Images and Personal Information. Proceedings of the 1st Transdisciplinary Conference on Distributed Diagnosis and Home Healthcare (D2H2).

[43] Ni B., Dat N.C., Moulin P. RGBD-Camera Based Get-Up Event Detection for Hospital Fall Prevention. Proceedings of the IEEE International Conference on Acoustics, Speech and Signal Processing (ICASSP 2012).

[44] Rougier C., Meunier J., St-Arnaud A., Rousseau J. (2011). Robust Video Surveillance for Fall Detection Based on Human Shape Deformation. IEEE Trans. Circuits Syst. Video Technol..

[45] Sixsmith A., Johnson N. (2004). A Smart Sensor to Detect the Falls of the Elderly. IEEE Pervasive Comput..

[46] Vishwakarma V., Mandal C., Sural S., Ghosh A., de Rajat K., Pal S.K. (2007). Automatic Detection of Human Fall in Video. Pattern Recognition and Machine Intelligence.

[47] Yu M., Naqvi S.M., Chambers J. A Robust Fall Detection System for the Elderly in a Smart Room. Proceedings of the IEEE International Conference on Acoustics Speech and Signal Processing (ICASSP 2010).

[48] Bagalà F., Becker C., Cappello A., Chiari L., Aminian K., Hausdorff J.M., Zijlstra W., Klenk J. (2012). Evaluation of Accelerometer-Based Fall Detection Algorithms on Real-World Falls. PLoS ONE.

[49] Gasparrini S., Cippitelli E., Spinsante S., Gambi E. (2014). A Depth-Based Fall Detection System Using a Kinect^®^ Sensor. Sensors.

[50] Hegde R., Sudarshan B., Kumar S., Hariprasad S., Satyanarayana B. (2013). Technical Advances in Fall Detection System—A Review. Int. J. Comput. Sci. Mob. Comput..

[51] Patsadu O., Nukoolkit C., Watanapa B. (2012). Survey of Smart Technologies for Fall Motion Detection: Techniques, Algorithms and Tools. Commun. Comput. Inf. Sci..

[52] Zhang T., Wang J., Liu P., Hou J. (2006). Fall Detection by Embedding an Accelerometer in Cellphone and using KFD Algorithm. Int. J. Comput. Sci. Netw. Secur..

[53] Sposaro F., Tyson G. IFall: An Android Application for Fall Monitoring and Response. Proceedings of the Annual International Conference of the IEEE Engineering in Medicine and Biology Society (EMBC 2009).

[54] Magaña-Espinoza P., Aquino-Santos R., Cárdenas-Benítez N., Aguilar-Velasco J., Buenrostro-Segura C., Edwards-Block A., Medina-Cass A. (2014). WiSPH: A Wireless Sensor Network-Based Home Care Monitoring System. Sensors.

[55] Zhang S., McCullagh P., Zhang J., Yu T. (2014). A Smartphone Based Real-Time Daily Activity Monitoring System. Clust. Comput..

[56] Chen M., Chiu Y., Chen C., Chen E. (2013). Implementation of Fall Detection and Localized Caring System. Math. Probl. Eng..

[57] Krenzel D., Warren S., Li K., Natarajan B., Singh G. Wireless Slips and Falls Prediction System. Proceedings of the Annual International Conference of the IEEE Engineering in Medicine and Biology Society (EMBC’2012).

[58] Pivato P., Dalpez S., Macii D., Petri D. A Wearable Wireless Sensor Node for Body Fall Detection. Proceedings of the IEEE International Workshop on Measurements and Networking Proceedings (M&N).

[59] Honglun H., Meimei H., Minghui W. (2013). Sensor-Based Wireless Wearable Systems for Healthcare and Falls Monitoring. Int. J. Smart Sens. Intell. Syst..

[60] Chang S., Lai C., Chao H.J., Park J.H., Huang Y. (2011). An Environmental-Adaptive Fall Detection System on Mobile Device. J. Med. Syst..

[61] Wang C., Liu C., Wang T. (2013). A Remote Health Care System Combining a Fall down Alarm and Biomedical Signal Monitor System in an Android Smart-Phone. Int. J. Adv. Comput. Sci. Appl..

[62] Yuan B., Herbert J. Non-Intrusive Movement Detection in Cara Pervasive Healthcare Application. Proceedings of the 2011 International Conference on Wireless Networks, (WORLDCOMP).

[63] Shuo C. Fall Detection System Using Arduino Fio. Proceedings of the IRC Conference on Science, Engineering and Technology.

[64] Pannurat N., Thiemjarus S., Nantajeewarawat E. (2014). Automatic Fall Monitoring: A Review. Sensors.

[65] IDC Worldwide Mobile Phone Tracker. http://www.idc.com/.

[66] Zhao Z., Chen Y., Liu J. Fall Detecting and Alarming Based on Mobile Phone. Proceedings of the 7th International Conference on Ubiquitous Intelligence & Computing and 7th International Conference on Autonomic & Trusted Computing (UIC/ATC 2010).

[67] Zhao Z., Chen Y., Wang S., Chen Z. FallAlarm: Smart Phone Based Fall Detecting and Positioning System. Proceedings of the 9th International Conference on Mobile Web Information Systems (MobiWIS).

[68] Laguna M.A., Finat J. Remote Monitoring and Fall Detection: Multiplatform Java Based Mobile Applications. Proceedings of the III International Workshop of Ambient Assisted (Living IWAAL 2011).

[69] Majumder A.J.A., Zerin I., Ahamed S.I., Smith R.O. (2014). A Multi-Sensor Approach for Fall Risk Prediction and Prevention in Elderly. ACM SIGAPP Appl. Comput. Rev..

[70] Allen B., Derveloy R., Fell N., Gasior W., Yu G., Sartipi M. Telemedicine Assessment of Fall Risk using Wireless Sensors. Proceedings of the 10th Annual IEEE Communications Society Conference on Sensor, Mesh and Ad Hoc Communications and Networks (SECON, 2013).

[71] Majumder A.J.A., Zerin I., Uddin M., Ahamed S.I., Smith R.O. SmartPrediction: A Real-Time Smartphone-Based Fall Risk Prediction and Prevention System. Proceedings of the 2013 Research in Adaptive and Convergent Systems (RACS 2013).

[72] Zhuang Y., Baldwin J., Antunna L., Yazir Y.O., Ganti S. Tradeoffs in Cross Platform Solutions for Mobile Assistive Technology. Proceedings of the IEEE Pacific Rim Conference on Communications, Computers and Signal Processing (PACRIM 2013).

[73] Shinde B.A., Chawan P.M. (2014). Dementia Patient Movement Detection and Fall Detection Using Smart Phone Technology. Int. J. Adv. Technol. Eng. Sci..

[74] Fontecha J., Navarro F.J., Hervás R., Bravo J. (2013). Elderly Frailty Detection by using Accelerometer-Enabled Smartphones and Clinical Information Records. Pers. Ubiquitous Comput..

[75] Clark M., Lim J., Tewolde G., Kwon J. (2015). Affordable Remote Health Monitoring System for the Elderly Using Smart Mobile Device. Sens. Transducers.

[76] Tacconi C., Mellone S., Chiari L. Smartphone-Based Applications for Investigating Falls and Mobility. Proceedings of the 5th International Conference on Pervasive Computing Technologies for Healthcare (PervasiveHealth 2011).

[77] Mazilu S., Hardegger M., Zhu Z., Roggen D., Troster G., Plotnik M., Hausdorff J.M. Online Detection of Freezing of Gait with Smartphones and Machine Learning Techniques. Proceedings of the 6th International Conference on Pervasive Computing Technologies for Healthcare (PervasiveHealth 2012).

[78] Otis M., Menelas B. Toward an Augmented Shoe for Preventing Falls Related to Physical Conditions of the Soil. Proceedings of the IEEE International Conference on Systems, Man, and Cybernetics (SMC’2012).

[79] Ko C., Leu F., Lin I., Xhafa F., Moore P., Tadros G. (2015). Using a Smartphone as a Track and Fall Detector: An Intelligent Support System. Advanced Technological Solutions for E-Health and Dementia Patient Monitoring.

[80] Guimaraes V., Teixeira P.M., Monteiro M.P., Elias D., Godara B., Nikita K.S. (2012). Phone Based Fall Risk Prediction. Wireless Mobile Communication and Healthcare.

[81] Oner M., Pulcifer-Stump J.A., Seeling P., Kaya T. Towards the Run and Walk Activity Classification through Step Detection-an Android Application. Proceedings of the Annual International Conference of the IEEE Engineering in Medicine and Biology Society (EMBC 2012).

[82] Yavuz G., Kocak M., Ergun G., Alemdar H.O., Yalcin H., Incel O.D., Ersoy C. (2010). A Smartphone Based Fall Detector with Online Location Support. Proceedings of the International Workshop on Sensing for App Phones.

[83] Kaladevi P., Kokila T., Narmatha S., Janani V. (2014). Accident Detection Using Android Smart Phone. Int. J. Innov. Res. Comput. Commun. Eng..

[84] Yu X. Approaches and Principles of Fall Detection for Elderly and Patient. Proceedings of the 10th International Conference on E-Health Networking, Applications and Services (HealthCom 2008).

[85] Noury N., Rumeau P., Bourke A., ÓLaighin G., Lundy J. (2008). A Proposal for the Classification and Evaluation of Fall Detectors. IRBM.

[86] Perry J.T., Kellog S., Vaidya S.M., Youn J., Ali H., Sharif H. Survey and Evaluation of Real-Time Fall Detection Approaches. Proceedings of the 6th International Symposium on High-Capacity Optical Networks and Enabling Technologies (HONET 2009).

[87] Hijaz F., Afzal N., Ahmad T., Hasan O. Survey of Fall Detection and Daily Activity Monitoring Techniques. Proceedings of the 2010 International Conference on Information and Emerging Technologies (ICIET).

[88] El-Bendary N., Tan Q., Pivot F.C., Lam A. (2013). Fall Detection and Prevention for the Elderly: A Review of Trends and Challenges. Int. J. Smart Sens. Intell. Syst..

[89] Delahoz Y.S., Labrador M.A. (2014). Survey on Fall Detection and Fall Prevention Using Wearable and External Sensors. Sensors.

[90] Monitoring of Human Movements for Fall Detection and Activities Recognition in Elderly Care Using Wireless Sensor Network: A Survey. http://www.intechopen.com/books/wireless-sensor-networks-application-centric-design/monitoring-of-human-movements-for-fall-detection-and-activities-recognition-in-elderly-care-using-wi.

[91] Schwickert L., Becker C., Lindemann U., Maréchal C., Bourke A., Chiari L., Helbostad J., Zijlstra W., Aminian K., Todd C. (2013). Fall Detection with Body-Worn Sensors: A Systematic Review. Z. Für Gerontol. Geriatr..

[92] Habib M.A., Mohktar M.S., Kamaruzzaman S.B., Lim K.S., Pin T.M., Ibrahim F. (2014). Smartphone-Based Solutions for Fall Detection and Prevention: Challenges and Open Issues. Sensors.

[93] Howcroft J., Kofman J., Lemaire E.D. (2013). Review of Fall Risk Assessment in Geriatric Populations Using Inertial Sensors. J Neuroeng. Rehabil..

[94] Castillo J.C., Carneiro D., Serrano-Cuerda J., Novais P., Fernández-Caballero A., Neves J. (2014). A Multi-Modal Approach for Activity Classification and Fall Detection. Int. J. Syst. Sci..

[95] Yi W., Saniie J. Design Flow of a Wearable System for Body Posture Assessment and Fall Detection with Android Smartphone. Proceedings of the IEEE International Technology Management Conference (ITMC).

[96] Wagner M., Kuch B., Cabrera C., Enoksson P., Sieber A. Android Based Body Area Network for the Evaluation of Medical Parameters. Proceedings of the 10th Workshop on Intelligent Solutions in Embedded Systems (WISES 2012).

[97] Kozlovszky M., Sicz-Mesziár J., Ferenczi J., Márton J., Windisch G., Kozlovszky V., Kotcauer P., Boruzs A., Bogdanov P., Meixner Z., Godara B., Nikita K.S. (2012). Combined Health Monitoring and Emergency Management through Android Based Mobile Device for Elderly People. Wireless Mobile Communication and Healthcare.

[98] Harbert S.D., Jaiswal T., Harley L.R., Vaughn T.W., Baranak A.S. Mobile Motion Capture-MiMiC. Proceedings of the 35th Annual International Conference of the IEEE Engineering in Medicine and Biology Society (EMBC 2013).

[99] Silva M., Teixeira P.M., Abrantes F., Sousa F., Gabrielli S., Elias D., Kahol K. (2011). Design and Evaluation of a Fall Detection Algorithm on Mobile Phone Platform. Ambient Media and Systems.

[100] Vogt P., Kuhn J. (2012). Analyzing Free Fall with a Smartphone Acceleration Sensor. Phys. Teach..

[101] Mehner S., Klauck R., Koenig H. Location-Independent Fall Detection with Smartphone. Proceedings of the ACM 6th International Conference on Pervasive Technologies Related to Assistive Environments (PETRA 2013).

[102] Mellone S., Tacconi C., Chiari L. (2012). Validity of a Smartphone-Based Instrumented Timed up and Go. Gait Posture.

[103] Albert M.V., Kording K., Herrmann M., Jayaraman A. (2012). Fall Classification by Machine Learning Using Mobile Phones. PLoS ONE.

[104] Faulkner M.N. (2014). Selective Data Gathering in Community Sensor Networks. Master’s Thesis.

[105] Medrano C., Igual R., Plaza I., Castro M. (2014). Detecting Falls as Novelties in Acceleration Patterns Acquired with Smartphones. PLoS ONE.

[106] Fudickar S., Lindemann A., Schnor B. Threshold-Based Fall Detection on Smart Phones. Proceedings of the 7th International Conference on Health Informatics (HEALTHINF’2014).

[107] Abbate S., Avvenuti M., Cola G., Corsini P., Light J., Vecchio A. Recognition of False Alarms in Fall Detection Systems. Proceedings of the 2011 IEEE Consumer Communications and Networking Conference (CCNC).

[108] Android Developers: Class Overview of SensorManager Class. http://developer.android.com/reference/android/hardware/SensorManager.html.

[109] Lockhart J.W., Pulickal T., Weiss G.M. Applications of Mobile Activity Recognition. Proceedings of the 2012 ACM Conference on Ubiquitous Computing.

[110] Dai J., Bai X., Yang Z., Shen Z., Xuan D. (2010). Mobile Phone-Based Pervasive Fall Detection. Pers. Ubiquitous Comput..

[111] Dai J., Bai X., Yang Z., Shen Z., Xuan D. PerFallD: A Pervasive Fall Detection System Using Mobile Phones. Proceedings of the 8th IEEE International Conference on Pervasive Computing and Communications Workshops (PERCOM Workshops).

[112] Brezmes T., Rersa M., Gorricho J., Cotrina J. Surveillance with Alert Management System Using Conventional Cell Phones. Proceedings of the 2010 Fifth International Multi-Conference on Computing in the Global Information Technology (ICCGI).

[113] Suh M., Chen C., Woodbridge J., Tu M.K., Kim J.I., Nahapetian A., Evangelista L.S., Sarrafzadeh M. (2011). A Remote Patient Monitoring System for Congestive Heart Failure. J. Med. Syst..

[114] Lee R.Y., Carlisle A.J. (2011). Detection of Falls Using Accelerometers and Mobile Phone Technology. Age Ageing.

[115] Chiu P.P.K., Lee T.K.S., Cheng J.M.F., Yeung S.T. Health Guard System with Emergency Call Based on Smartphone. Proceedings of the IET International Communication Conference on Wireless Mobile and Computing (CCWMC 2011).

[116] Teixeira P., Nunes F., Silva P., Texeira L. Mover-Activity Monitor and Fall Detector for Android. Proceedings of the Mobile Wellness Workshop at Mobile HCI 2011 (MW2011).

[117] Viet V., Choi D. Fall Detection with Smart Phone Sensor. Proceedings of the 3rd International Conference on Internet (ICONI 2011).

[118] Viet V.Q., Lee G., Choi D. Fall Detection Based on Movement and Smart Phone Technology. Proceedings of the IEEE RIVF International Conference on Computing and Communication Technologies, Research, Innovation, and Vision for the Future (RIVF 2012).

[119] Hsieh S., Su M.H., Liu L.F., Jiang W. A Finite State Machine-Based Fall Detection Mechanism on Smartphones. Proceedings of the 9th International Conference on Ubiquitous Intelligence & Computing and 9th International Conference on Autonomic & Trusted Computing (UIC/ATC 2012).

[120] A Self Organizing Map Based Motion Classifier with an Extension to Fall Detection Problem and its Implementation on a Smartphone. http://www.intechopen.com/books/applications-of-self-organizing-maps/a-self-organizing-map-based-motion-classifier-with-an-extension-to-fall-detection-problem-and-its-im.

[121] Thammasat E., Chaicharn J. A Simply Fall-Detection Algorithm Using Accelerometers on a Smartphone. Proceedings of the 5th Biomedical Engineering International Conference (BMEiCON 2012).

[122] Abbate S., Avvenuti M., Bonatesta F., Cola G., Corsini P., Vecchio A. (2012). A Smartphone-Based Fall Detection System. Pervasive Mob. Comput..

[123] Uegami M., Iwamoto T., Matsumoto M. A Study of Detection of Trip and Fall using Doppler Sensor on Embedded Computer. Proceedings of the 2012 IEEE International Conference on Systems, Man, and Cybernetics (SMC).

[124] Fang S., Liang Y., Chiu K. Developing a Mobile Phone-Based Fall Detection System on Android Platform. Proceedings of the Computing, Communications and Applications Conference (ComComAp).

[125] Cao Y., Yang Y., Liu W.H. E-FallD: A Fall Detection System using Android-Based Smartphone. Proceedings of the 9th International Conference on Fuzzy Systems and Knowledge Discovery (FSKD 2012).

[126] He Y., Li Y., Bao S. Fall Detection by Built-in Tri-Accelerometer of Smartphone. Proceedings of the IEEE EMBS International Conference onBiomedical and Health Informatics (BHI 2012).

[127] He Y., Li Y., Yin C. (2012). Falling-Incident Detection and Alarm by Smartphone with Multimedia Messaging Service (MMS). E-Health Telecommun. Syst. Netw..

[128] Shi Y., Shi Y., Wang X. Fall Detection on Mobile Phones using Features from a Five-Phase Model. Proceedings of the 9th International Conference on Ubiquitous Intelligence & Computing and 9th International Conference on Autonomic & Trusted Computing (UIC/ATC 2012).

[129] Hwang S., Ryu M., Yang Y., Lee N. Fall Detection with Three-Axis Accelerometer and Magnetometer in a Smartphone. Proceedings of the International Conference on Computer Science and Technology (CST 2012).

[130] Megalingam R.K., Unnikrishnan D.K.M., Radhakrishnan V., Jacob D.C. HOPE: An Electronic Gadget for Home-Bound Patients and Elders. Proceedings of the 2012 Annual IEEE India Conference (INDICON 2012).

[131] Pavlakis P., Alepis E., Virvou M. Intelligent Mobile Multimedia Application for the Support of the Elderly. Proceedings of the 8th International Conference on Intelligent Information Hiding and Multimedia Signal Processing (IIH-MSP 2012).

[132] Fahmi P.N., Viet V., Deok-Jai C. Semi-Supervised Fall Detection Algorithm using Fall Indicators in Smartphone. Proceedings of the 6th International Conference on Ubiquitous Information Management and Communication (ICUIMC'2012).

[133] Mellone S., Tacconi C., Schwickert L., Klenk J., Becker C., Chiari L. (2012). Smartphone-Based Solutions for Fall Detection and Prevention: The FARSEEING Approach. Z. Für Gerontol. Geriatr..

[134] Hou Y., Li N., Huang Z. Triaxial Accelerometer-Based Real Time Fall Event Detection. Proceedings of the International Conference on Information Society (i-Society).

[135] Kim Y., Kim S., Kang D., Park H., Kim N., Yang S.H., Kim Y. A Simple Falling Recognition Scheme for a Human Body by using Mobile Devices. Proceedings of the 1st International Conference on Advanced Information and Computer Technology (AICT).

[136] Boehner A. A Smartphone Application for a Portable Fall Detection System. Proceedings of the National Congress of Undergraduate Research (NCUR).

[137] Terroso M., Freitas R., Gabriel J., Marques A.T., Simoes R. Active Assistance for Senior Healthcare: A Wearable System for Fall Detection. Proceedings of the 8th Iberian Conference Onformation Systems and Technologies (CISTI 2013).

[138] Chou W., Lin W., Lee M., Lei K.F. Design and Assessment of a Real-Time Accelerometer-Based Lying-to-Sit Sensing System for Bed Fall Prevention. Proceedings of the IEEE International Conference on Systems, Man, and Cybernetics (SMC).

[139] Horta E.T., Lopes I.C., Rodrigues J.J.P.C., Proenca M.L. A Mobile Health Application for Falls Detection and Biofeedback Monitoring. Proceedings of the IEEE 15th International Conference on e-Health Networking, Applications and Services (Healthcom 2013).

[140] Koshmak G.A., Linden M., Loutfi A. Evaluation of the Android-Based Fall Detection System with Physiological Data Monitoring. Proceedings of the 35th Annual International Conference of the IEEE Engineering in Medicine and Biology Society (EMBS, 2013).

[141] Tan T., Gochoo M., Chang C., Wu C., Chiang J.Y. Fall Detection for Elderly Persons Using Android-Based Platform. Proceedings of the 8th International Forum on Strategic Technology (IFOST 2013).

[142] Wibisono W., Arifin D.N., Pratomo B.A., Ahmad T., Ijtihadie R.M. Falls Detection and Notification System using Tri-Axial Accelerometer and Gyroscope Sensors of a Smartphone. Proceedings of the 2013 Conference on Technologies and Applications of Artificial Intelligence (TAAI).

[143] Cabestany J., Moreno J.M., Perez C., Sama A., Catala A., Rodriguez-Molinero A., Arnal M. FATE: One Step Towards an Automatic Aging People Fall Detection Service. Proceedings of the 20th International Conference Mixed Design of Integrated Circuits and Systems (MIXDES).

[144] Zhang Q., Ren L., Shi W. (2013). HONEY: A Multimodality Fall Detection and Telecare System. Telemed. E-Health.

[145] Megalingam R.K., Pocklassery G., Mourya G., Prabhu R.M., Jayakrishnan V., Kanhiroth V., Katoch R., Veliyara P.S. Measurement of Elder Health Parameters and the Gadget Designs for Continuous Monitoring. Proceedings of the 3rd International Conference on Advancements in Electronics and Power Engineering (ICAEPE'2013).

[146] Yang B., Lee Y., Lin C. Time Fall Detecting and Protecting System Using Mobile Device. Proceedings of the International Conference on Fall Prevention and Protection.

[147] Serafimov K., Koceska N. (2013). Pervasive Alert System for Fall Detection Based on Mobile Phones. Yearbook of the Faculty of Computer Science.

[148] He Y., Li Y. (2013). Physical Activity Recognition Utilizing the Built-in Kinematic Sensors of a Smartphone. Int. J. Distrib. Sens. Netw..

[149] Horta E.T., Lopes I.C., Rodrigues J.J., Misra S. Real Time Falls Prevention and Detection with Biofeedback Monitoring Solution for Mobile Environments. Proceedings of the IEEE 15th International Conference One-Health Networking, Applications & Services (Healthcom 2013).

[150] Kansiz A.O., Guvensan M.A., Turkmen H.I. Selection of Time-Domain Features for Fall Detection Based on Supervised Learning. Proceedings of the World Congress on Engineering and Computer Science.

[151] Lee J., Chuah Y., Chieng K.T. (2013). Smart Elderly Home Monitoring System with an Android Phone. Int. J. Smart Home.

[152] Tiwari R., Singh A.K., Khan S.N. Using Android Platform to Detect Free Fall. Proceedings of the 2013 International Conference on Information Systems and Computer Networks (ISCON).

[153] Shen V.R., Lai H., Lai A. Application of High-Level Fuzzy Petri Nets to Fall Detection System Using Smartphone. Proceedings of the International Conference on Machine Learning and Cybernetics (ICMLC 2013).

[154] Shen V.R., Lai H., Lai A. (2015). The Implementation of a Smartphone-Based Fall Detection System using a High-Level Fuzzy Petri Net. Appl. Soft Comput..

[155] Mao L., Liang D., Ning Y., Ma Y., Gao X., Zhao G. Pre-Impact and Impact Detection of Falls using Built-in Tri-Accelerometer of Smartphone. Proceedings of the Third International Conference on Health Information Science (HIS 2014).

[156] Kau L., Chen C. A Smart Phone-Based Pocket Fall Accident Detection, Positioning and Rescue System. Proceedings of IEEE International Symposium on Bioelectronics and Bioinformatics (ISBB).

[157] Aguiar B., Rocha T., Silva J., Sousa I. Accelerometer-Based Fall Detection for Smartphones. Proceedings of the IEEE International Symposium on Medical Measurements and Applications (MeMeA).

[158] Zhang Y., Ning H., Bai J., Chen B., Zhou P., Zhao X. Elderly Safety Early-Warning System Based on Android Mobile Phones. Proceedings of the 10th International Conference on Natural Computation (ICNC).

[159] Rudraraju T. (2014). Elderly Support-Android Application for Fall Detection and Tracking. Master’s Thesis.

[160] Maglogiannis I., Ioannou C., Spyroglou G., Tsanakas P., Iliadis L., Maglogiannis I.L., Papadopoulos (2014). Fall Detection Using Commodity Smart Watch and Smart Phone. Artificial Intelligence Applications and Innovations.

[161] Medrano C., Igual R., Plaza I., Castro M., Fardoun H. Personalizable Smartphone Application for Detecting Falls. Proceedings of the IEEE-EMBS International Conference on Biomedical and Health Informatics (BHI).

[162] Dau H.A., Salim F.D., Song A., Hedin L., Hamilton M. Phone Based Fall Detection by Genetic Programming. Proceedings of the 13th International Conference on Mobile and Ubiquitous Multimedia.

[163] Ferreira A.B., Piva L.S., Braga R.B., de Castro Andrade R.M. Trust Evaluation in an Android System for Detection and Alert Falls. Proceedings of the 20th Brazilian Symposium on Multimedia and the Web.

[164] Luque R., Casilari E., Morón M., Redondo G. (2014). Comparison and Characterization of Android-Based Fall Detection Systems. Sensors.

[165] De Maso-Gentile G., Malizia S., Basili L., Orcioni S., Pirani S., Conti M. Low Power Fall Detection System. Proceedingd of the 6th European Conference of the International Federation for Medical and Biological Engineering (IFMBE’2105).

[166] Salgado P., Afonso P. Body Fall Detection with Kalman Filter and SVM. Proceedings of the 11th Portuguese Conference on Automatic Control (CONTROLO’201).

[167] Kerdegari H., Mokaram S., Samsudin K., Ramli A.R. (2015). A Pervasive Neural Network Based Fall Detection System on Smart Phone. J. Ambient Intell. Smart Environ..

[168] Dinh T., Chew M. Application of a Commodity Smartphone for Fall Detection. Proceedings of the 6th International Conference on Automation, Robotics and Applications (ICARA 2015).

[169] Mulcahy M.K. Automatic Fall Detection using Mobile Devices. Proceedings of the 46th ACM Technical Symposium on Computer Science Education.

[170] Rasheed M.B., Javaid N., Alghamdi T.A., MuNhtar S., Qasim U., Khan Z.A., Raja M.H.B. Evaluation of Human Activity Recognition and Fall Detection Using Android Phone. Proceedings of the 2015 IEEE 29th International Conference on Advanced Information Networking and Applications.

[171] Soewito B. (2015). Medical Alert System using Fall Detection Algorithm on Smartphone. Int. J. Softw. Eng. Appl..

[172] Huq G., Maeder A., Basilakis J., Pirnejad H. Trialling a Personal Falls Monitoring System using Smart Phone. Proceedings of the 8th Australasian Workshop on Health Informatics and Knowledge Management (HKIM 2015).

[173] Thiemjarus S., Henpraserttae A., Marukatat S. A Study on Instance-Based Learning with Reduced Training Prototypes for Device-Context-Independent Activity Recognition on a Mobile Phone. Proceedings of the 2013 IEEE International Conference on Body Sensor Networks (BSN).

[174] Zhuang X., Huang J., Potamianos G., Hasegawa-Johnson M. Acoustic Fall Detection Using Gaussian Mixture Models and GMM Supervectors. Proceedings of the IEEE International Conference on Acoustics, Speech and Signal Processing (ICASSP 2009).

[175] Yang X., Dinh A., Che L. A Wearable Real-Time Fall Detector Based on Naive Bayes Classifier. Proceedings of the 23rd Canadian Conference on Electrical and Computer Engineering (CCECE 2010).

[176] Majumder A.J.A., Rahman F., Zerin I., Ebel W., Ahamed S.I. IPrevention: Towards a Novel Real-Time Smartphone-Based Fall Prevention System. Proceedings of the 28th Annual ACM Symposium on Applied Computing.

[177] Tra K., Pham T.V. Human Fall Detection Based on Adaptive Background Mixture Model and HMM. Proceedings of the 2013 International Conference on Advanced Technologies for Communications (ATC).

[178] Kangas M., Konttila A., Winblad I., Jamsa T. Determination of Simple Thresholds for Accelerometry-Based Parameters for Fall Detection. Proceedings of the 29th Annual International Conference of the IEEE Engineering in Medicine and Biology Society (EMBS 2007).

[179] Kangas M., Konttila A., Lindgren P., Winblad I., Jämsä T. (2008). Comparison of Low-Complexity Fall Detection Algorithms for Body Attached Accelerometers. Gait Posture.

[180] Bao L., Intille S.S., Ferscha A., Mattern F. (2004). Activity Recognition from User-Annotated Acceleration Data. Pervasive Computing.

[181] Gibson M.J., Andres R.O., Isaacs B., Radebaugh T., Wormpetersen J. (1987). The Prevention of Falls in Later Life-A Report of the Kellogg-International-Work-Group on the Prevention of Falls by the Elderly. Dan. Med. Bull..

[182] Becker C., Schwickert L., Mellone S., Bagalà F., Chiari L., Helbostad J., Zijlstra W., Aminian K., Bourke A., Todd C. (2012). Proposal for a Multiphase Fall Model Based on Real-World Fall Recordings with Body-Fixed Sensors. Z. Für Gerontol. Geriatr..

[183] Bai Y., Wu S., Yu C.H. Recognition of Direction of Fall by Smartphone. Proceedings of the 26th Annual IEEE Canadian Conference on Electrical and Computer Engineering (CCECE 2013).

[184] Vavoulas G., Pediaditis M., Spanakis E.G., Tsiknakis M. The MobiFall Dataset: An Initial Evaluation of Fall Detection Algorithms using Smartphones. Proceedings of the IEEE 13th International Conference on Bioinformatics and Bioengineering (BIBE 2013).

[185] Weiss A., Shimkin I., Giladi N., Hausdorff J.M. (2010). Automated Detection of Near Falls: Algorithm Development and Preliminary Results. BMC Res. Notes.

[186] Klenk J., Becker C., Lieken F., Nicolai S., Maetzler W., Alt W., Zijlstra W., Hausdorff J., van Lummel R., Chiari L. (2011). Comparison of Acceleration Signals of Simulated and Real-World Backward Falls. Med. Eng. Phys..

[187] Kangas M. (2011). Development of Accelerometry-Based Fall Detection. Ph.D. Thesis.

[188] Kangas M., Vikman I., Nyberg L., Korpelainen R., Lindblom J., Jämsä T. (2012). Comparison of Real-Life Accidental Falls in Older People with Experimental Falls in Middle-Aged Test Subjects. Gait Posture.

[189] Joe J., Demiris G. (2013). Older Adults and Mobile Phones for Health: A Review. J. Biomed. Inform..

[190] Human Computer Intelligent Interaction Lab (HCII-Lab, Guangzhou, China): SCUT-NAA Dataset. http://www.hcii-lab.net/data/scutnaa/EN/naa.html.

[191] Xue Y., Jin L. A Naturalistic 3D Acceleration-Based Activity Dataset & Benchmark Evaluations. Proceedings of the IEEE International Conference on Systems Man and Cybernetics (SMC’2010).

[192] Telecommunication System Team (TST) TST Fall Detection DataBase. http://www.tlc.dii.univpm.it/blog/databases4kinect#IDFall.

[193] MEI Research Actipal, 16 January 2014. http://www.meinergy.com.

[194] Kerdegari H., Samsudin K., Ramli A.R., Mokaram S. Evaluation of Fall Detection Classification Approaches. Proceedings of the 4th International Conference on Intelligent and Advanced Systems (ICIAS 2012).

[195] Google Play Store. https://play.Google.com/store.

[196] Klenk J., Chiari L., Helbostad J., Zijlstra W., Aminian K., Todd C., Bandinelli S., Kerse N., Schwickert L., Mellone S. (2013). Development of a Standard Fall Data Format for Signals from Body-Worn Sensors. Z. Für Gerontol. Geriatr..

[197] Fawcett T. (2006). An Introduction to ROC Analysis. Pattern Recog. Lett..

[198] Viet V.Q. (2013). Trading off Accuracy and Energy Consumption in Activity Recognition on Mobile Phones. Master’s Thesis.

[199] Viet V.Q., Negera A.F.P., Thang H.M., Choi D. (2013). Energy Saving in Forward Fall Detection Using Mobile Accelerometer. Int. J. Distrib. Syst. Technol..

[200] Wang W. (2013). Supporting Fall Prevention for the Elderly by Using Mobile and Ubiquitous Computing. Master’s Thesis.

[201] Kangas M., Korpelainen R., Vikman I., Nyberg L., Jamsa T. (2015). Sensitivity and False Alarm Rate of a Fall Sensor in Long-Term Fall Detection in the Elderly. Gerontology.

[202] Wireless Medical Technologies: Navigating Government Regulation in the New Medical Age. http://www.fr.com/files/Uploads/attachments/FinalRegulatoryWhitePaperWirelessMedicalTechnologies.pdf.

